# Hippo signalling pathway mediates oncogenic properties of NAB2::STAT6 in solitary fibrous tumour

**DOI:** 10.1007/s13402-026-01173-x

**Published:** 2026-02-10

**Authors:** Carmen Salguero-Aranda, Laura Lobo-Selma, Amparo Beltrán-Povea, Sergio Baute-González, Paula Gilabert-Prieto, Carmen Jordán-Perez, José Alberto Fernández-Juárez, Ana Teresa Amaral, René Rodríguez, Juan Díaz-Martín, Enrique de Álava

**Affiliations:** 1 Instituto de Biomedicina de Sevilla, Department of Pathology, CSIC-Universidad de Sevilla, Hospital Universitario Virgen del Rocío, Seville, Spain; 2 Centro de Investigación Biomédica en Red de Cáncer, Instituto de Salud Carlos III, Madrid, Spain; 3 Tumour Microenvironment and Targeted Therapies Group, CiBB-Center for Innovation in Biomedicine and Biotechnology, Universidade de Coimbra, Coimbra, Portugal; 4 Sarcomas and Experimental Therapeutics, Instituto de Investigación Sanitaria del Principado de Asturias (ISPA), Oviedo, 33011 Spain; 5 Instituto Universitario de Oncología del Principado de Asturias (IUOPA), Oviedo, 33011 Spain; 6 Instituto de Biomedicina y Biotecnología de Cantabria (IBBTEC), Universidad de Cantabria-CSIC, Santander, Spain; 7 Department of Normal and Pathological Cytology and Histology, School of Medicine, University of Seville, Seville, Spain

**Keywords:** Solitary fibrous tumour, Gene fusion, NAB2:STAT6, Hippo signalling pathway, Dasatinib

## Abstract

**Purpose:**

Solitary fibrous tumour (SFT) is a rare mesenchymal neoplasm molecularly defined by the *NAB2::STAT6* gene fusion (GF), an aberrant transcriptional regulator whose functions beyond *EGR1* activation remain incompletely understood. This study aimed to further elucidate the oncogenic role of *NAB2::STAT6* and to identify potential therapeutic vulnerabilities.

**Methods:**

Human mesenchymal stem cell–derived SFT models (SFT-MSCs) were generated via ectopic expression of *NAB2::STAT6* and analysed by gene expression microarray to assess the impact of this fusion on the transcriptomic profile. Stable SFT-MSC clones were subjected to functional assays following *YAP1/TAZ* silencing via siRNAs or pharmacological inhibition with dasatinib. Transcriptomic profiling of 16 tumours was performed to investigate correlations between Hippo pathway and EGR1 transcriptional signatures. Nuclear YAP1/TAZ expression was assessed by immunohistochemistry (IHC) in 44 patient samples, and genomic structural variations (SVs) were analyzed in 8 SFT specimens through Optical Genome Mapping.

**Results:**

*NAB2::STAT6* ectopic expression led to Hippo pathway dysregulation and promoted a malignant phenotype, which was partially reversible upon *YAP1/TAZ* knockdown or dasatinib treatment. Total RNA-seq of SFT local cases confirmed transcriptional inactivation of Hippo signalling and revealed a network linking Hippo and EGR1 pathways. Stronger nuclear YAP1/TAZ staining was observed in relapsed SFT samples compared with primary tumors. The overall genomic stability precluded Hippo pathway deregulation via SVs in clinical samples.

**Conclusion:**

NAB2::STAT6 promotes SFT progression by inactivating the Hippo pathway, unveiling a potential targetable vulnerability and further expanding our understanding of NAB2::STAT6-driven oncogenesis.

**Supplementary Information:**

The online version contains supplementary material available at 10.1007/s13402-026-01173-x.

## Introduction

Solitary fibrous tumour (SFT) is a rare and ubiquitous mesenchymal neoplasm with an incidence of 1 new case per million people per year, representing fewer than 4% of all adult soft tissue sarcomas and mesenchymal tumours [1–3]. Clinically, SFT typically presents as a painless mass in adult patients, most commonly between 20 and 70 years of age, with no gender predilection [4]. This neoplasm has an intermediate malignant potential and a metastasis risk of 35–45%, or even greater, in series with extended follow-up [5, 6]. Modern risk-stratification models, such as that of *Demicco et al.* [7], have replaced the traditional “typical” and “malignant” categories for prognostic assessment, yet a subset of patients remains difficult to classify, highlighting the need for additional molecular markers [8–13].

The molecular hallmark of SFT is a recurrent inversion at chromosomal region 12q13 that generates a gene fusion (GF) between NGFI-A-binding protein 2 (*NAB2*) and signal transducer and activator of transcription 6 (*STAT6*), *NAB2::STAT6*, the primary oncogenic driver [14, 15]. Despite the substantial variability in the GF breakpoint, the resulting chimeric protein consistently loses at least one repressor domain from NAB2, a well-established co-repressor of *Early Growth Response 1* (EGR1), which is replaced by a transactivation domain from STAT6. This structural rearrangement leads to constitutive activation of EGR1-mediated transcription and establishes a feed-forward transcriptional loop that promotes neoplastic progression [14–16].

Early studies demonstrated that the fusion deregulates the EGR1 pathway and upregulates several receptor tyrosine kinases (RTKs) [15], prompting clinical trials evaluating RTK inhibitors in advanced SFT [4, 17–22]. Although these agents provided some clinical benefit, responses were variable and generally modest, underscoring the need for more effective approaches. Subsequent profiling studies have shown that this dysregulated EGR1 axis drives upregulation of downstream targets, thereby promoting proliferative and angiogenic phenotypes in SFT [23–25]. Although the *NAB2::STAT6* fusion and its link to the EGR1 axis are well established as defining features of SFT, the broader biological implications of the fusion are largely unexplored. This is partly due to the limited availability of SFT-derived cell lines and patient-derived xenograft (PDX) models, which have been used almost exclusively for evaluating therapeutic compounds [25–28].

To address this challenge, we established MSC-derived SFT models expressing NAB2::STAT6, followed by transcriptomic and functional analyses. Exploratory analyses revealed deregulation of the Hippo signalling pathway, prompting us to focus subsequent investigations on this pathway. By integrating in vitro experiments with patient-derived samples, this study examines how NAB2::STAT6-associated programmes intersect with Hippo signalling and EGR1 networks, providing a rationale for further exploration of Hippo modulation as a potential therapeutic avenue.

## Materials and methods

### Cell culture

Immortalized hMSCs harbouring 5 oncogenic hits (hMSC-5 H) were previously developed and used for sarcoma modelling [29, 30]. hMSC-5 H were cultured in Dulbecco´s modified Eagle´s medium (DMEM) with Glutamax supplemented with 10% fetal bovine serum and 1% penicillin/streptomycin (all from Gibco, Thermo Fisher Scientific) and routinely tested for mycoplasma infection. Cells were grown in vitro at 37 °C and 5% CO_2_.

Mammalian gene expression lentiviral vectors for ectopic expression of *NAB2::STAT6* GFs (*NAB2ex4::STAT6ex2* and *NAB2ex6::STAT6ex16* GF variants) were acquired from *VectorBuilder*. mCherry and Bsd linked by T2A were introduced to allow cells (SFT-MSC) to be visualised by red fluorescence and to be resistant to blasticidin, respectively. An empty vector without GF cDNA was employed as a control to generate c-MSC. hMSC-5 H cells were transfected with jetPRIME transfection reagent (Polyplus) using a ratio 1:1 (plasmid: reagent), according to the manufacturer´s instructions. Cells were harvested three days after transfection to assess GF expression by qPCR and western blotting (WB). Furthermore, transfected cells were cultured as single cells in 96-well plates to generate cell clones by cell sorting in a BD FACSAria™ Fusion flow cytometer (BD Bioscience). Positive clones overexpressing the GF *NAB2ex6::STAT6ex16* were confirmed by fluorescence microscopy and flow cytometry, thanks to the mCherry reporter gene incorporated in the vector and two of them, identified as SFT-MSC #1 and SFT-MSC #2, were used for further experiments.

### Cell line RNA extraction, retrotranscription and qPCR

Total RNA from the cell lines was extracted using the miRNeasy kit (QIAGEN). Total RNA was quantified with a Nanodrop ND-2000 Spectrophotometer. At least, 500 ng was used for reverse transcription using the high-capacity cDNA reverse transcription kit (Thermo Fisher Scientific) according to the manufacturer’s instructions. qPCR was performed with TaqMan™ Universal PCR Master Mix (Applied Biosystems) and the TaqMan probes (Thermo Fisher Scientific) detailed in the Supplementary Table 1. Data from qRT-PCR studies were analysed with S.D.S. (Sequence Detection System version 2.4). The fold change was calculated by using the *ΔΔCt* algorithm and c-MSC was considered the reference sample.

### Protein extraction and western blot analysis

Total protein lysis was performed on ice using RIPA buffer supplemented with protease and phosphatase inhibitors (Roche) and two cycles of 30 s sonication were done using Bioruptor sonicator (Diagenode). Protein quantification was analysed by Pierce BCA Protein Assay Kit (Thermo Fisher Scientific) according to the manufacturer´s instructions using the microplate reader TECAN infinite M200 (TECAN). Nucleus and cytoplasm subcellular fractionations were performed to identify the subcellular location of the proteins of interest. Briefly, cytoplasmic lysis buffer (250 mM sucrose, 50 mM Tris-HCl pH 4.7, 0.25% NP40, supplemented with protease and phosphatase inhibitors) was added to the cells, incubated for 10 min on ice and cells were centrifuged at 500 g for 5 min. Cytoplasmic fraction-containing supernatant was recovered. After a wash step (1 M sucrose, 50 mM Tris-HCI pH 7.4, 5 mM MgCl_2_), the nuclear lysis buffer (0.4 M NaCI, 20 mM Tris-HCI pH 7.4, 15% v/v glycerol, 1.5% v/v triton X-100, supplemented with protease and phosphatase inhibitors) was added to the cells and two cycles of 30 s sonication were performed using Bioruptor sonicator (Diagenode). Finally, cells were centrifuged at 5000 g 4 °C for 3 min and the nuclear fraction-containing supernatant was isolated. Protein quantification was done as detailed for total protein lysis. Immunoblot detection was carried out using SuperSignal West Pico PLUS Chemiluminescent Substrate (Thermo Fisher Scientific) and visualised by digital imaging in a Chemidoc Touch Imaging System (Bio-Rad). Quantification of WB was performed using the ImageJ software 1.53j (National Institutes of Health, Bethesda, MD, USA). The antibodies used in this study are detailed in the Supplementary Table 1. Three independent biological samples were analysed, unless otherwise specified in the figure legends.

### Microarray analysis

Total RNA from SFT-MSC and c-MSC cell lines was extracted and assessed for quality as described in Sect. 2.2. The human transcriptome was evaluated using the Clariom^™^ S Human array (Thermo Fisher Scientific). Briefly, 100 ng of total RNA was amplified and labeled using the GeneChip^®^ WT PLUS Reagent Kit (Thermo Fisher Scientific, Inc.) following procedures described in the Kit user manual. The amplified cDNA was quantified, fragmented, and labeled in preparation for hybridization to the GeneChip^®^ Clariom S Human Array (Thermo Fisher Scientific, Inc.) using 5.5 µg of single-stranded cDNA and following protocols defined in the user manual. Washing, staining (GeneChip^®^ Fluidics Station 450, Thermo Fisher Scientific, Inc.), and scanning (GeneChip^®^ Scanner 3000, Thermo Fisher Scientific, Inc.) were performed following protocols outlined in the user manual for cartridge arrays. The analysis was conducted in three biological replicates of each sample. CELL files were analysed with the Transcriptome Analysis Console (TAC) 4.0 software (Thermo Fisher Scientific), which performs statistical analysis and provides a list of differentially expressed genes (DEGs). Fold change > 1.5 and P-value < 0.05 were set as cut-off values. Gene Set Enrichment Analysis (GSEA v4.3.2) was conducted to identify biological pathways and transcription factors potentially regulating significantly upregulated genes. CEL files from four well-annotated clinical samples generated in the study by *Hajdu M. et al.* [31] (Human Genome U133A array platform) were retrieved and integrated with our transcriptomic data obtained from the mesenchymal model. Raw microarray data from the two independent assays were processed in R using *RStudio* [32]. Background correction, normalization, and summarization were performed using the Robust Multi-array Average (RMA) method as implemented in the *oligo* package [33]. Probe-level data were mapped to ENTREZ Gene identifiers using the *AnnotationDbi* package, retaining only probes with unambiguous gene annotations for downstream analyses [34]. DEGs were identified using the *limma* package [35] based exclusively on the Clariom S microarray samples. Genes with a nominal p-value < 0.05 and an absolute fold change ≥ 1.5 were considered significantly differentially expressed. The resulting DEG set was used for downstream exploratory analyses, including dimensionality reduction by t-distributed stochastic neighbor embedding (t-SNE) implemented in the *Rtsne* package [36], as well as hierarchical clustering using Euclidean distance and Ward.D2 linkage, as implemented in the *dist* and *hclust* functions in R. When U133A samples were incorporated, datasets were integrated by retaining the intersection of genes common to both platforms. Batch effects associated with microarray platform differences were corrected using the *ComBat* method from the *sva* package [37]. Data visualization was performed using the *ggplot2* [38] and *plotly* [39] packages.

### Tumour sample selection

Tumour tissue samples were obtained from the HUVR-IBiS Biobank (Virgen del Rocio University Hospital –Institute of Biomedicine of Seville, Andalusian Public Health System Biobank). Two expert pathologists evaluated fresh or formalin-fixed, paraffin-embedded (FFPE) samples. Clinical diagnosis of all the samples was performed by morphological and immunohistochemical testing, with special focus on the evaluation of STAT6 expression. In total, 44 SFT samples were evaluated from 38 patients, comprising 31 primary tumours and 13 relapsed tumours. For 4 primary tumour samples, paired relapsed tumours were available. For one patient, first and second relapsed tumours were retrieved. In addition, the presence of the *NAB2::STAT6* GF was confirmed by targeted RNA-seq (as detailed in our previous publication) [13] and/or total RNA-seq (detailed below) in 30 out of 38 patients. This study was in accordance with standard Spanish ethical regulations and was approved by the institutional ethics committee of the Virgen del Rocio University Hospital of Seville, Spain. Written informed consent was obtained from all patients and all clinical analyses were conducted in accordance with the principles of the Declaration of Helsinki.

### YAP1/TAZ immunohistochemistry

Two different tissue microarrays (TMA) were constructed using FFPE material. We analysed 41 FFPE samples from 35 different SFT patients. Specifically, 28 samples corresponded to primary tumours, and 13 samples were relapsed tumours. YAP1/TAZ IHC was performed on TMA sections using the Envision method (Dako, CA, USA), with a heat-induced antigen-retrieval step, and a primary antibody against YAP1 and TAZ (supplementary Table 1). YAP1/TAZ nuclear staining was evaluated by an experienced pathologist, who assigned staining intensity scores (0-1-2-3), and % area. The total staining score (TSS) was calculated as the product of intensity and area, ranging from 0 to 300. Median TSS value was set to classify cases as “low” or “high” for clinical correlation analysis. Medical records and clinicopathologic information were retrospectively reviewed by biobank.

### Optical genome mapping

Biobanked OCT-frozen material from sixteen patients diagnosed with SFT between 2010 and 2023 at Virgen del Rocío University Hospital (HUVR) was included in the study. Hematoxylin–eosin slides were reviewed by an expert pathologist to confirm the presence of at least 50% tumour cells. Tissue samples of approximately 10 mg were sectioned into 10-µm slices and processed by *Bionano Genomics* at its facilities. In brief, high molecular weight DNA (UHMW DNA) fragments (> 150 Kbp) were extracted using the Bionano Prep SP Tissue and Tumour Kit (Bionano Genomics), which relies on paramagnetic nanodiscs to reduce DNA fragmentation. After homogenization, DNA was quantified with the Qubit Broad Range dsDNA (Invitrogen™) and labelled using a Direct Label and Stain DNA Labelling Kit where the DLE-1 enzyme targeted the CTTAAG motif without introducing strand breaks, allowing the generation of genome maps of 20–100 Mbp. Eight samples did not meet UHMW DNA quality/quantity metrics and were excluded from the analysis. Labeled DNA was loaded onto Saphyr 3.3 chips to reach a minimum yield of 320 Gbp. Molecules were electrophoretically linearized, imaged, and digitally aligned to the GRCh38 reference genome. Data quality was evaluated based on DNA fragment length, mapping efficiency, coverage depth, label density, and label variance. Bioinformatic analyses were executed on Bionano Solve V3.8 using two pipelines: Rare Variant Analysis (RVA) and De Novo Assembly and reporting and direct visualization of structural variations (SVs) were done on Bionano Access v1.7.

### Total RNA-sequencing

Sixteen frozen OCT conserved samples were selected following evaluation by expert pathologists. Total RNA was isolated from 10 μm tissue sections using the miRNeasy Micro Kit (QIAGEN), in accordance with the manufacturer’s instructions, with the assistance of the ULTRA-TURRAX^®^ to improve cell lysis efficiency. DNase treatment was applied to eliminate the genomic DNA. Extracted RNA was quantified using the fluorometer Qubit™ 4 (Invitrogen™), according to the manufacturer´s protocol. The integrity of the RNA was evaluated by TAPE Station (Agilent). All of them passed the RNA quality control thresholds (RIN > 2 and DV200 > 55). 100 ng from total RNA was used for the library, using Illumina Stranded Total RNA Prep, Ligation with Ribo-Zero Plus (Illumina), according to the manufacturer’s instructions. Libraries were quantified using the Qubit™ 4 fluorometer (Invitrogen™). The flow cell used was the SP Reagent Kit v1.5 (200 cycles) 2*100 bp to run in a single batch the 16 SFT samples, with a total of 50 million of reads per sample in the sequencer NovaSeq6000 (Illumina). Raw data were analysed using RNASeq pipeline from *nf-core* with default parameters [40]. The integrated web application iDEP was used for the differential expression and pathway analysis of RNA-Seq data [41]. To identify GF transcripts, BaseSpace Sequence Hub (Illumina, Illumina Inc., San Diego, CA) was used in combination with Arriba [42], FusionCatcher [43], and STAR-Fusion [44], requiring concordant detection by at least three tools to confirm the presence of a GF. Normalized gene expression matrix was analysed using single-sample Gene Set Enrichment Analysis (ssGSEA) implemented in the *GSEAPy* package. Cordenonsi_YAP_conserved_signature and EGR1_01 were provided as a GMT file and used to compute Normalized Enrichment Scores (NES) for each sample. NES values were used directly without additional scaling or normalization. The association between both signatures was quantified using Pearson correlation coefficient. All analyses and graphical representations were performed in Python using *pandas*, *scipy*, *matplotlib*, and *seaborn* libraries.

### Cell morphological assays

Cell morphology was evaluated through bright light and fluorescent microscopy images in a fluorescence microscopy (BX-61, Olympus). Calcein-acetoxymethyl ester (Calcein-AM) reagent (Thermo Fisher Scientific) was added to the cells according to the manufacturer´s instructions. Changes in cell morphology were also measured by flow cytometry graphing the size (forward scatter, FSC) and complexity (side scatter, SSC) in a countour plot, in a BD LSRFortessa flow cytometer (BD Bioscience). Three independent experiments were done, unless otherwise specified in the figure legends.

### Cell cycle

For cell cycle analysis, 60% confluent cells were fixed using 70% ice-cold ethanol, permeabilized with a solution of phosphate-buffered saline (PBS) with 0.1% Triton X100 and stained with 4´,6-diamino-2-phenylindole (DAPI) (10 µg/mL) (Thermo Fisher Scientific), for DNA labelling. At least 20,000 cells were analysed per sample from three independent experiments in a BD LSRFortessa flow cytometer (BD Bioscience). Cell cycle statistics were obtained with FlowJo™ v10.6.1 Software (BD Life Sciences).

### Cell proliferation in 2D

1,000 cells per well were seeded in 96-well plates, with five replicates per condition. After 6 days of cell culture, MTT assay (Roche) was used following manufacturer’s instructions. Absorbance of the colourimetric reaction was measured at 565 nm by using a microplate reader, TECAN Infinite M200 (TECAN).

### Spheroid formation

Spheroid generation was performed by culturing 1,000 cells per well into a 96-well Clear Round Bottom Ultra-Low Attachment Microplate (Corning, Life Sciences). Spheroid volumes were monitored using an inverted microscope (IX-71, Olympus) and bright-field images were taken at day 7. Between 6 and 8 replicates were cultured per condition. Diameter of the spheroids was measured in triplicated by ImageJ software 1.53j (National Institutes of Health, Bethesda, MD, USA). Scale bar length was used to convert arbitrary units of length to µm. Spheroid volumes were calculated with the sphere volume formula (V = 4/3*π*r^3^) and indicated in the figures as 10^6^ µm^3^.

### Wound healing

Wound-healing assays were performed with confluent cells, and wounds were created in triplicate using a pipette tip. Images of the same selected area at initial time (T0) and after 6 h (T6) were taken using an inverted microscope (IX-71, Olympus). The percentage of migratory cells was calculated by measuring the area at T0 and T6 using the ImageJ software version 1.53j. At least, three independent experiments were done for all the assays.

### Immunofluorescence (IF)

10^4^ cells were seeded on coverslips in 24-well plates. When cells reached 60–70% confluence (after 3–4 days of cell culture), slides were washed twice with PBS, then fixed with 4% PFA for 10 min at room temperature (RT), and washed with 125 mM glycine for 15 min at RT. After two additional washes, cells were permeabilized with PBS-0.1% Triton X-100 for 30 min at RT and blocked with PBS-2% BSA for 1 h at RT, and incubated with primary antibodies in PBS-1% BSA overnight at 4°C (Supplementary Table 1). Slides were washed twice with PBS and incubated with the corresponding secondary antibody (Supplementary Table 1) in PBS-1% BSA for 1 h at RT. After washes, cells were counterstained with DAPI for 10 min at RT and mounted using DAKO Fluorescent Mounting Medium. Images were acquired using Olympus BX61 Fluorescence Motorized Microscope. To determine the nuclear intensity, a mask was generated using DAPI and the “analyze particle” tool of ImageJ software 1.53j. Then, mask was applied to the IF images. Mean values relativized to control condition are represented in the graph.

### siRNA treatment

Silencing of *YAP1*, *WWTR1* and *EGR1* was performed by using 10 or 20 nM of ON-target plus siRNAs (Horizon), whose sequences are detailed in the Supplementary Table 1. Cells were transfected with jetPRIME transfection reagent (Polyplus) according to the manufacturer´s instructions. Twenty-four hours after transfection, cells were harvested to measure expression at the RNA level, and to perform in vitro growth assays. Forty-eight hours post-transfection, the expression of target genes was evaluated by WB. A siRNA designed against a non-human coding sequence (non-targeting control, NT) was used as negative control in all assays.

### Dasatinib treatment

To determine the half-maximal inhibitory concentration (IC50) of dasatinib (#9052, Cell Signalling Technology), 3,000 cells were seeded per well in 96-well plates. Twenty-four hours later, cells were treated with increasing concentrations of dasatinib and incubated for 72 h. Cell viability was assessed using the MTT assay (Roche), following the manufacturer’s instructions. Dose–response curves and IC50 values were calculated using GraphPad Prism software. The IC50 for GF-expressing clones was subsequently used in all downstream experiments.

### Statistics

Statistical significance of in vitro functional assays was evaluated using a two-tailed Student’s t-test or a Mann-Whitney U test for parametric or nonparametric data, respectively, comparing SFT-MSC model data with c-MSC data as the control. Correlation between immunohistochemistry (IHC) YAP1/TAZ expression and clinicopathological characteristics was assessed by Fisher’s exact test for the categorical variables. The Mann–Whitney test was used for the analysis of differences in the continuous variables. All these statistical analyses were performed using GraphPad Prism v7 (GraphPad Software, San Diego, CA, USA). *p*-value < 0.05 was considered statistically significant.

## Results

### Transcriptional activity driven by *NAB2::STAT6* correlates with the Hippo signalling pathway signature

**Fig. 1:**
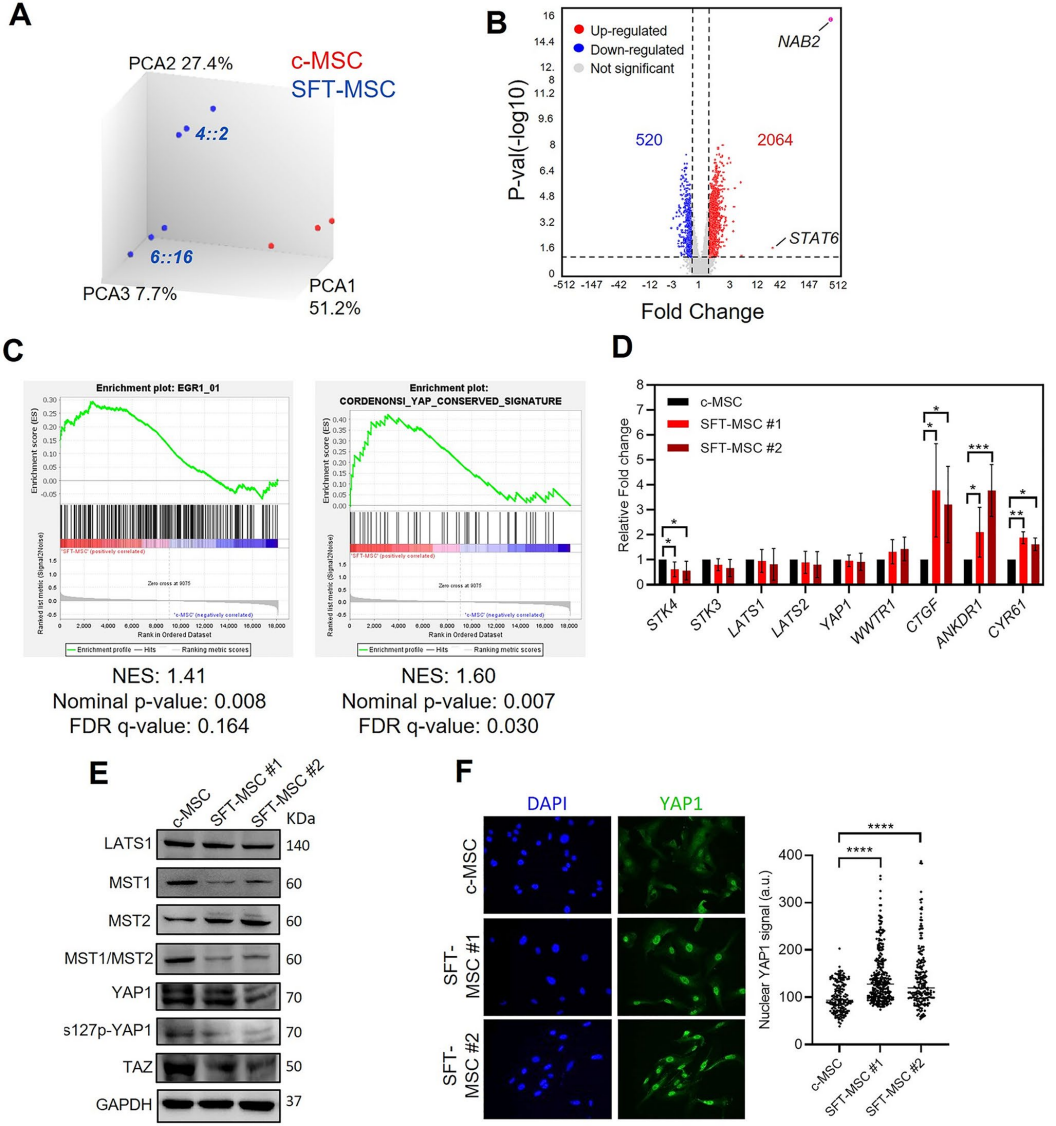
NAB2::STAT6-driven transcriptional activity correlates with Hippo signalling. **(A)** Principal component analysis (PCA) of c-MSC (red) and SFT-MSC (blue) samples illustrating transcriptomic differences. **(B)** Volcano plot depicting differential gene expression between c-MSC and SFT-MSC groups. Significant genes are highlighted. **(C)** Gene Set Enrichment Analysis (GSEA) comparing c-MSC (reference) and SFT-MSC. Left: enrichment plot for transcription factor EGR1 targets. Right: enrichment plot for the oncogenic pathway *Cordenonsi YAP conserved signature*. NES: Normalized Enrichment Score. Nominal *P*-value and FDR *q*-value are shown. **(D)** qPCR analysis of Hippo pathway–related genes. GAPDH was used as endogenous control; c-MSC served as the reference condition. **(E)** Western blots for Hippo pathway proteins. GAPDH was used as a loading control. Data represent at least three independent experiments. **(F)** Immunofluorescence analysis of YAP1/TAZ. Left: representative images showing DAPI (blue) and YAP1/TAZ (green), 40x objective. Right: nuclear YAP1/TAZ quantification from two independent experiments normalized to c-MSCs; mean values are indicated by horizontal bars

While the exact cellular origin of SFT has not been definitively established, MSCs are widely considered plausible precursor cells [4]. Accordingly, we used immortalized hMSCs (hMSC-5 H), previously employed to model other sarcomas via ectopic GF expression [29], to overexpress the two most common GF variants in SFTs, *NAB2ex4::STAT6ex2* and *NAB2ex6::STAT6ex16* [13]. As a control condition (c-MSC), cells were transfected with an empty vector. After confirming GF expression at mRNA and protein levels (Supplementary Fig. 1A, B), we performed gene expression profiling using Affymetrix microarrays. Unsupervised hierarchical clustering segregates c-MSC and SFT-MSC cells (Supplementary Fig. 1C), and PCA showed sample distribution based on the presence of the GF versus the control vector, while also separating the cells according to the two GF variants (Fig. 1A). To examine whether our mesenchymal model faithfully recapitulates the transcriptional landscape of actual tumours, we integrated transcriptomic data from several well-annotated, publicly available SFT clinical samples [31] with our mesenchymal model data. Remarkably, clinical samples clustered closely with the SFT-MSC models and segregated according to the *NAB2::STAT6* fusion variant (Supplementary Fig. 1D, E), highlighting that the mesenchymal context provides a robust platform for studying transcriptional regulation and pathway dynamics in SFT. Differential gene expression analysis revealed 2064 genes significantly up-regulated in cells transfected with both GFs compared with the control cells, including *NAB2* (310.61-fold) and *STAT6* (24.3-fold), and 520 genes significantly down-regulated (Fig. 1B) (Supplementary Table 2). Next, this transcriptional profile was compared with previously published curated gene sets. Motif enrichment analysis revealed a significant enrichment of EGR1 transcription factor binding motif in genes up-regulated in the SFT-MSC models, which confirms the central role of EGR1 in the deregulation of gene expression induced by the GF. Interestingly, Gene Set Enrichment Analysis (GSEA) revealed significant enrichment of *Cordenonsi YAP conserved* signature in SFT-MSC cells (Fig. 1C).

To confirm the deregulation of this pathway, we generated stable SFT-MSC clones. Two clones harbouring the *NAB2ex6::STAT6ex16* GF variant (SFT-MSC #1 and SFT-MSC #2) were successfully established and randomly selected based on their total STAT6 protein expression compared with control cells. However, stable clones harbouring *NAB2ex4::STAT6ex2* could not be obtained, which could be attributable to the larger cDNA insert. These models were first confirmed to express the GF by flow cytometry (Cherry-positive) and subcellular WB, demonstrating that expression of the GF recapitulates the STAT6 nuclear localisation observed in SFT (Supplementary Fig. 1F, G). The expression of the GF also leads to notable morphological changes, characterized by increased cell size and higher granularity. These alterations were consistently observed through both flow cytometry, which revealed enhanced forward and side scatter signals, and microscopic analysis (Supplementary Fig. 1H, I). Furthermore, we analysed the expression of EGR1 target genes to validate that our model faithfully recapitulates the EGR1 pathway activation characteristic of SFT. Notably, *FGF2* and *FGFR1*, two canonical EGR1 target genes, were significantly induced in SFT-MSC cells (Supplementary Fig. 1J). The assessment of the Hippo-core related genes by qPCR showed that *STK4* (also known as *MST1*, mammalian sterile 20-like kinase 1), a serine/threonine kinase that serves as a core component of the Hippo signalling pathway, is significantly reduced in SFT-MSC models compared to c-MSC. This result is in agreement with data from the microarray assay (FC, -1.68; FDR P-value 0.0414) (Supplementary Table 2). Remarkably, well-known Hippo target genes such as *CTGF*, *ANKDR1* and *CYR61* were significantly increased in cells expressing the GF compared to control, indicating that the GF promotes the Hippo signalling pathway inactivation (Fig. 1D). Moreover, WB analysis confirmed a reduction in MST1/STK4 protein levels and its phosphorylated form, as well as a decrease in the inactive form of YAP1 (S127p-YAP1) (Fig. 1E). In the same context, immunofluorescence assays revealed that nuclear YAP1 signal was increased in SFT-MSC models compared to c-MSC (Fig. 1F).

Taken together, these findings suggest that the GF expression may be associated with reduced *STK4* expression, potentially contributing to decreased inhibitory phosphorylation of YAP1 at S127 and increased nuclear accumulation of active YAP1. This was accompanied by upregulation of canonical Hippo target genes (*CTGF*, *ANKRD1*, and *CYR61*), suggesting that the GF inactivates the Hippo pathway and enhances YAP1-driven transcriptional activity.

### The NAB2::STAT6-induced aggressive phenotype is partially abrogated upon YAP1/TAZ and EGR1 silencing

**Fig. 2:**
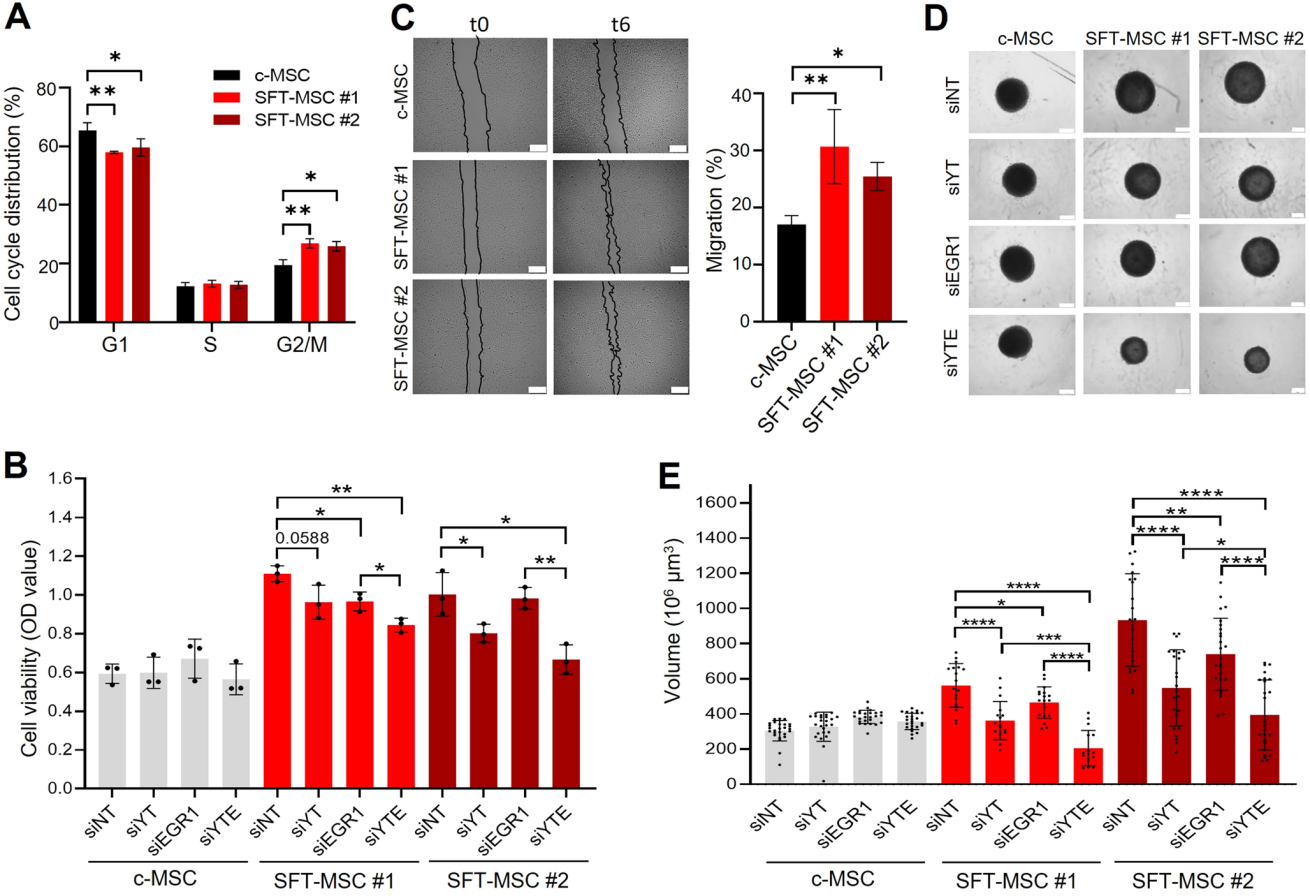
YAP1/TAZ silencing attenuates NAB2::STAT6-driven proliferation and stemness in SFT-MSCs. **(A)** Cell cycle phase distribution analysed by flow cytometry. **(B)** MTT assay quantification of cell viability after 7 days in cell models treated with siRNAs. All graphs represent mean ± SD of at least three independent experiments. **(C)** Cell migration evaluated by wound-healing assay. Left: representative images at 0 and 6 h; right: quantification of migration rates. **(D)** Representative spheroid images after 7 days under spheroid-promoting conditions and siRNA treatment (scale bar = 200 μm). **(E)** Spheroid volume quantification after 7 days in siRNA-treated cells. Each dot represents an individual spheroid; bars indicate mean ± SD. All experiments were performed at least in triplicate

Next, phenotypic and functional assays were performed to characterize our SFT-MSC models and elucidate the contribution of the Hippo signalling pathway. Our results showed that ectopic expression of the GF induced notable changes in cell cycle dynamics. SFT-MSC models showed a reduction in the G1 phase and an increase in the G2/M phase compared with control cells (Fig. 2A), indicating that the GF promotes a more proliferative state. Consistently, viability assays demonstrated that SFT-MSC proliferate faster than c-MSC under 2D culture conditions (unpaired t test, c-MSC vs. SFT-MSC #1; P value < 0.01; and c-MSC vs. SFT-MSC #2; P-value < 0.05) (Fig. 2B). Interestingly, wound healing assays revealed that GF expression enhances migratory capacity (Fig. 2C). Furthermore, in 3D culture assays, SFT-MSC formed larger spheroids than control cells (unpaired t test, c-MSC vs. SFT-MSC #1 and #2; P-value < 0.05) (Fig. 2D, E), suggesting increased stemness capacity. Collectively, these findings indicate that the GF drives the aggressive proliferative and migratory behavior of SFT-MSC.

We therefore sought to investigate the potential contribution of Hippo signalling to the aggressive phenotype of SFT-MSC. Thus, we used siRNAs to silence the Hippo pathway final effectors *YAP1* and *TAZ*. In parallel, silencing of *EGR1*—the key transcription factor implicated in SFT biology—was performed as a control. Interestingly, co-silencing of *YAP1/TAZ* and *EGR1* produced a more pronounced reduction in EGR1 expression than EGR1 silencing alone, suggesting potential cross-regulation among them. In addition, *YAP1/TAZ* silencing resulted in downregulation of the target genes *CTGF* and *ANKDR1.* No effect on the expression levels of *YAP1*,* TAZ*, or their target genes was observed upon EGR1 silencing (Supplementary Fig. 2A). Notably, siRNA treatment targeting either *YAP1/TAZ* or *EGR1* impaired both proliferation and sphere-forming capacity of SFT-MSCs, whereas control MSCs remained unaffected. Strikingly, simultaneous silencing of all three produced a pronounced synergistic suppression (Fig. 2B, D, E). However, the migratory capacity of SFT-MSC was not reduced after the treatment (Supplementary Fig. 2B), implying that additional pathways contribute to this phenotype. These findings suggest functional cooperation between *EGR1* and *YAP1/TAZ* in maintaining the proliferative/stemness potential of fusion-positive cells.

### Dasatinib selectively impairs proliferation of *NAB2::STAT6*-positive cells

To further explore the potential role of Hippo signalling in SFT, we pharmacologically inhibited this pathway in our GF-overexpressing cellular models using dasatinib, a tyrosine kinase inhibitor previously reported to block nuclear localisation of YAP/TAZ in other tumour contexts [45–47].

**Fig. 3:**
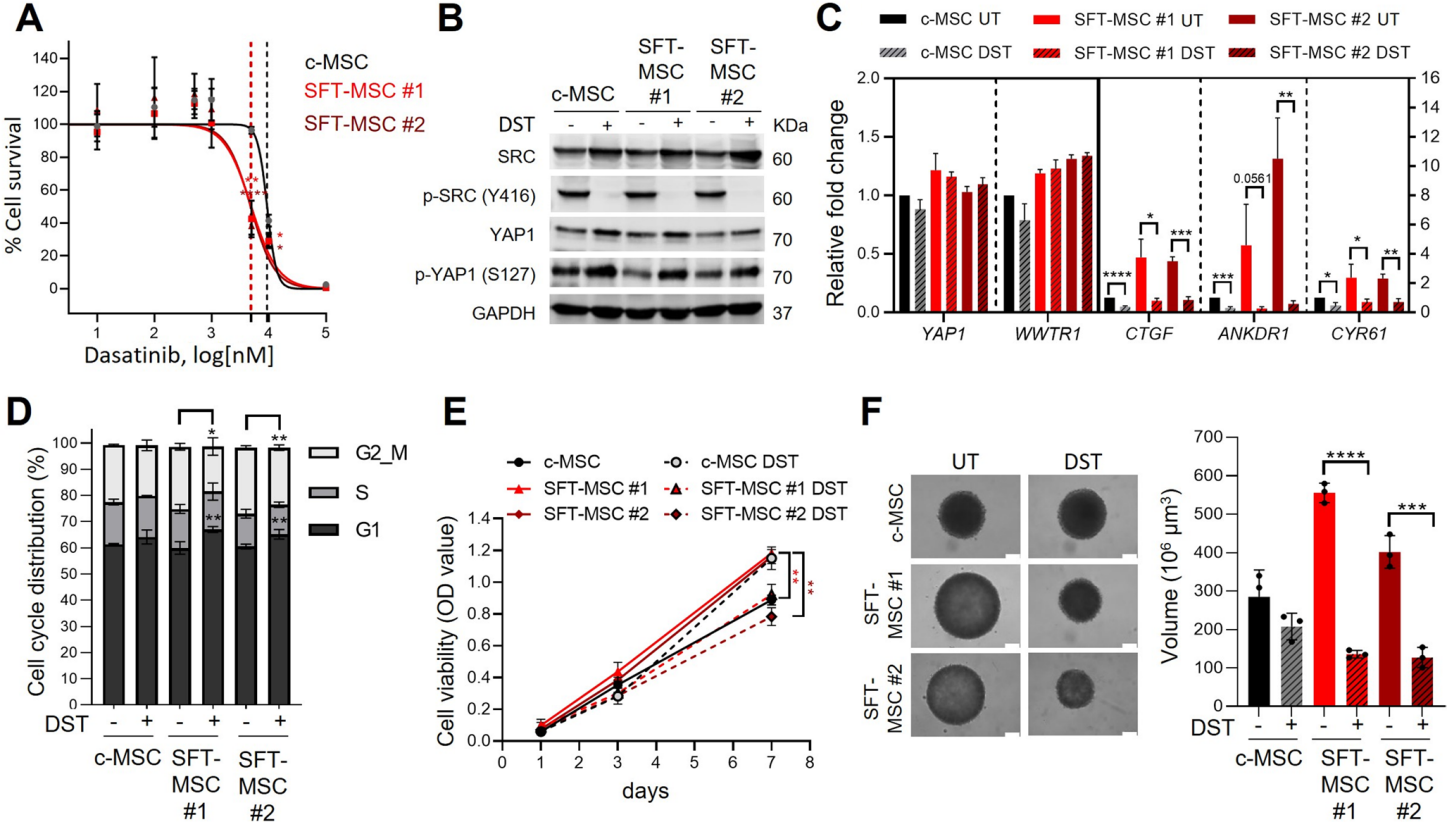
Dasatinib inhibits Hippo signalling pathway and selectively reduces proliferation in SFT-MSC. **(A)** Dose–response curves of c-MSC and SFT-MSC cells treated with dasatinib. IC₅₀ values indicated by vertical bars (black: c-MSC; light red/dark red: SFT-MSC). **(B)** Western blot analysis of SRC, YAP1, and phosphorylated SRC (Y416) and YAP1 (S127) after dasatinib treatment. GAPDH was used as a loading control. **(C)** qPCR analysis of Hippo pathway target genes (*CTGF*, *ANKRD1*, *CYR61*) after dasatinib exposure. Expression levels were normalized to *GAPDH* and compared with untreated c-MSCs. **(D)** Flow cytometry analysis of cell cycle phase distribution after dasatinib treatment. **(E)** MTT assay quantifying cell viability after 7 days of dasatinib treatment. **(F)** Spheroid formation assay after 7 days of dasatinib treatment. Left: spheroid volume quantification; right: representative spheroid images. All data are presented as mean ± SD of at least three independent experiments. Abbreviations: UT: untreated; DST: Dasatinib

Dose-response experiments revealed that SFT-MSC were significantly more sensitive to dasatinib than c-MSC (c-MSC: IC₅₀ = 9.39 µM; SFT-MSC #1: IC₅₀ = 4.53 µM; SFT-MSC #2: IC₅₀ = 4.82 µM) (Fig. 3A), leading us to select 5 µM for subsequent experiments. Mechanistically, WB analysis confirmed that dasatinib markedly reduced activating SRC phosphorylation (p-SRC Y416) while increasing inhibitory phosphorylation of YAP1 (p-YAP S127) (Fig. 3B). Furthermore, qPCR analysis showed a significant downregulation of canonical *YAP1/TAZ* target genes (*CTGF*, *ANKRD1*, and *CYR61*) upon treatment (Fig. 3C). Functionally, dasatinib induced G1-phase cell cycle arrest and a corresponding decrease in G2/M-phase cells in SFT-MSC (Fig. 3D). In 2D proliferation assays, dasatinib selectively suppressed SFT-MSC growth to levels comparable with c-MSC (Fig. 3E), while in 3D assays, it markedly impaired sphere-forming capacity of SFT-MSC models (Fig. 3F).

Overall, these findings indicate that dasatinib sensitivity is likely associated with the presence of the fusion protein, suggesting that this molecular alteration contributes to the therapeutic response.

### Multi-level characterization of the Hippo pathway in SFT patient samples

To further study the deregulation of the Hippo signalling pathway, we sought to perform total RNA-seq in 16 patient samples from our SFT series (Supplementary Table 3). We detected the *NAB2::STAT6* fusion in all analysed samples, while the reciprocal *STAT6::NAB2* fusion was observed in 50% of cases (8/16). Based on the conserved exons of STAT6, cases were further classified as FULL (STAT6 fully retained) or TAD (only the transactivation domain [TAD] of STAT6 retained), for further analysis. Furthermore, we observed that several cases harboured additional secondary GFs beyond *NAB2::STAT6* (Supplementary Table 4).

**Fig. 4:**
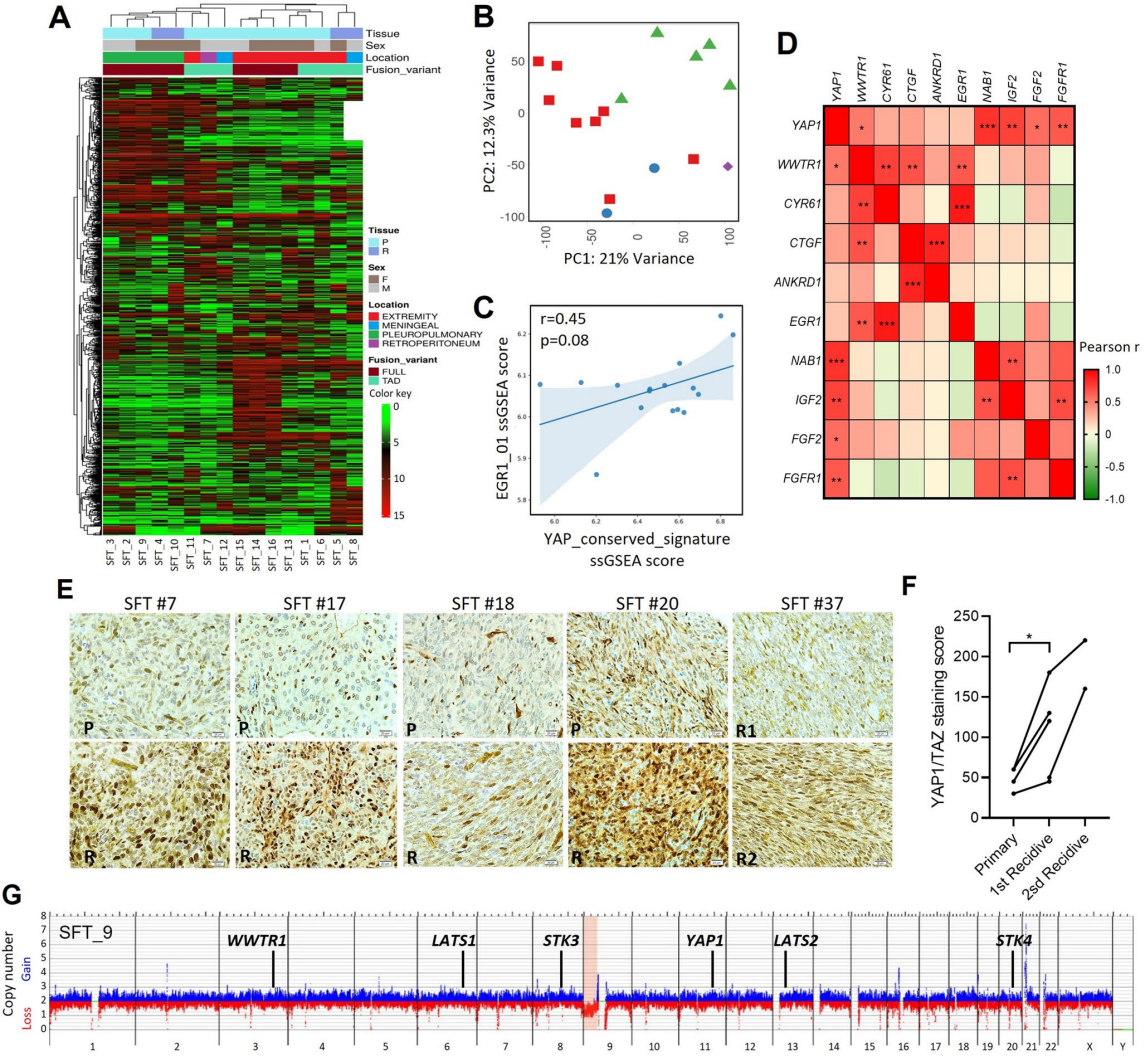
Multi-level characterization of Hippo pathway activation and its association with *NAB2::STAT6* fusion and *EGR1* expression in SFT patient samples. **(A)** Hierarchical clustering of 16 SFT cases based on total RNA-seq data. The heatmap depicts the top 1,000 differentially expressed genes (Pearson correlation). **(B)** Principal component analysis (PCA) of 16 SFT samples based on RNA-seq data. Distinct tumour locations are indicated by symbols/colors; Red: Extremity; Blue: Meningeal; Green: Pleuropulmonary; and Purple: Retroperitoneum. **(C)** Correlation analysis between Cordenonsi YAP conserved signature and EGR1 NES scores. Pearson’s correlation coefficient (r) and corresponding significance value are shown. **(D)** Heatmap of the correlation matrix computed using Pearson’s correlation coefficient (r) for every pair of data sets. Hippo signalling pathway genes and EGR1 target genes were included in the analysis. The colour gradient indicates the value of r, and statistically significant correlations are marked with one or more asterisks according to their level of statistical significance. **(E)** Representative immunohistochemical images of YAP1/TAZ in paired primary and recurrent SFT cases (60X objective). **(F)** Quantification of YAP1/TAZ nuclear staining scores in paired samples from primary tumours and their first and second recurrences. **(G)** Copy number variation (CNV) profile (gain: blue; loss: red) of SFT case #9 analysed by optical genome mapping (OGM). Core Hippo pathway members are highlighted. Abbreviations: P, primary; R, recurrent; R1: first relapse; R2: second relapse; F, female; M, male

Unsupervised hierarchical clustering segregated tumours from distinct anatomical locations (Fig. 4A, B). In contrast, no clear grouping was observed between primary and relapsed tumours or according to GF variant or patient gender. Regarding Hippo pathway, we assessed Hippo pathway activity in SFT cases through the valuation of *Cordenonsi YAP conserved* signature. The results showed that this signature is activated in SFTs, concomitant with EGR1 signature enrichment, with both exhibiting high ssGSEA NES scores and a positive correlation trend, suggesting coordinated pathway activity despite not reaching statistical significance (Fig. 4C, and Supplementary Fig. 3). Moreover, we investigated the correlations between the expression levels of the final effectors of the Hippo pathway—*YAP1* and *WWTR1*—and their well-described target genes, as well as EGR1 and its known downstream targets. Remarkably, *WWTR1*, but not *YAP1*, correlated with its own target genes as well as with *EGR1*, whereas *YAP1* displayed a strong positive correlation with several of EGR1’s known target genes (Fig. 4D).

We next assessed YAP1/TAZ expression, a surrogate marker of Hippo pathway deregulation, by IHC in 41 FFPE tumour samples from 35 SFT patients (28 primary and 13 relapsed tumours). We observed no significant associations with any clinical parameters when patients were grouped attending to the median TSS (Supplementary Fig. 4A). However, TSS data analysis as continuous variable revealed an association between nuclear YAP1/TAZ expression levels and patients´ age (age < 55 years: mean TSS = 97.6 ± 68.0 vs. age ≥ 55 years: mean TSS = 158.6 ± 65.6; Mann-Whitney U test, p-value 0.0068). Moreover, this analysis showed a trend toward an association between nuclear YAP1/TAZ levels and tumour location, although it did not reach statistical significance (Extrapleural: mean TSS = 145.9 ± 73.5 vs. Pleural: mean TSS = 88.8 ± 60.9, unpaired t-test, p-value 0.0554) (Supplementary Fig. 4B). Notably, four paired primary–relapse samples displayed increased nuclear YAP1/TAZ TSS in the relapsed tumour, with a comparable elevation observed in second-relapse pairs, suggesting that elevated YAP1/TAZ activity may be associated with more aggressive and therapy-resistant tumour phenotypes (Fig. 4E, F). However, no significant differences in TSS were observed between primary and recurrent samples across the cohort, likely due to substantial patient-to-patient heterogeneity (Supplementary Fig. 4C).

To investigate whether the deregulation of the Hippo pathway arises solely from transcriptional alterations, or whether genomic changes may also contribute to it, a cohort of 16 fresh SFT patient samples was analysed using OGM. Unfortunately, eight cases did not meet the quality criteria for UHMW DNA yield or integrity. The data from the remaining samples (Supplementary Table 5) revealed a low prevalence of large SVs (copy number variation [CNV], loss of heterozygosity [LOH] or aneuploidies) (Fig. 4G, Supplementary Fig. 5A), but multiple smaller insertions, deletions, and duplications distributed across the genome (Supplementary Fig. 5B, Supplementary Table 6). OGM data revealed a rearrangement between the *NAB2* and *STAT6* loci (Supplementary Fig. 5C) in 5 out of 8 cases (62.5%) when the *de novo* analysis was applied—a much higher detection rate compared to RVA, which identified the fusion in only 1 of 8 cases (12.5%). The poor performance of RVA for GF detection is due to the low enzyme labelling density within the *NAB2* locus, making this SV particularly difficult to detect using the RVA pipeline. OGM analysis of clinical SFT samples revealed a structurally simple and stable genome, with no additional large-scale SVs beyond the *NAB2::STAT6* fusion. No recurrent structural DNA alterations were detected, indicating that SFTs are characterized by limited genomic complexity. This simplicity extends to genes associated with the Hippo pathway, which were also unaffected by SVs, indicating that pathway deregulation in SFT is not attributable to genomic alterations. Interestingly, SFT_7 showed several SVs involving the *STAT6* locus (Supplementary Fig. 5D), suggesting that the GF was not formed by a simple inversion but rather through a more complex rearrangement. In line with these findings, total RNA-seq analysis identified multiple additional STAT6-related GFs in SFT_7, suggesting that the oncogenic fusion arises from a complex rearrangement event (Supplementary Table 4).

## Discussion

SFT is a rare mesenchymal neoplasm that typically follows an indolent and slow-growing clinical course. However, its intermediate malignant potential cannot be reliably predicted based solely on histopathological findings. Current risk models, such as the *Demicco* scoring system [7]—which considers age, tumour size, mitotic count, and necrosis—assist in estimating prognosis but still show limitations in accurately classifying all cases [48]. Recent research aims to refine these models by incorporating molecular alterations [9, 10, 13, 49, 50], although none has yet reached clinical application. The lack of effective systemic treatments for advanced disease and the poor survival rates of metastatic SFT, less than 50% at five years [48, 51], emphasizes the urgent need for better prognostic tools and targeted therapies.

Since the discovery of the *NAB2::STAT6* fusion as the principal oncogenic driver in SFT over a decade ago [15, 52], progress in elucidating its biological mechanisms has been limited, and effective therapeutic strategies remain lacking. This is partly attributable to the lack of preclinical models and the almost exclusive focus on drug testing. Early efforts focused mainly on PDX of dedifferentiated SFT, which enabled testing of conventional chemotherapeutics such as doxorubicin, dacarbazine (DTIC), ifosfamide (monotherapy or combination), trabectedin, and eribulin, with only modest efficacy observed [53]. More recently, several SFT cell lines derived from PDXs have been developed, facilitating the systematic testing of targeted drugs and drug combinations [25, 27, 28].

Beyond EGR1 deregulation, our findings—together with emerging evidence from others [23, 54, 55]—suggest that *NAB2::STAT6* exerts a broader transcriptional influence. Transcriptomic profiling of hMSC expressing *NAB2::STAT6* revealed profound remodeling of gene expression. Motif enrichment confirmed deregulation of *EGR1* target genes, supporting the notion of *EGR1* mediating the *NAB2::STAT6* induced transcriptional output [15, 23, 55, 56]. To functionally validate these mechanisms, we generated a stable hMSC line expressing the *NAB2ex6::STAT6ex16* fusion—one of the most prevalent variants in SFT [13]. Expression of this fusion induced profound phenotypic changes, including enhanced proliferation, accelerated cell cycle progression, greater spheroid formation, and increased migratory capacity. These results reinforce the oncogenic role of *NAB2::STAT6* in driving an aggressive cellular phenotype [15, 56].

Mechanistically, our findings show that expression of the *NAB2::STAT6* GF is associated with marked downregulation of *STK4 (MST1)*, a core kinase of the Hippo signalling pathway. This is particularly noteworthy, as loss or suppression of Hippo kinases has frequently been reported in sarcomas through post-translational regulation or epigenetic silencing [57, 58]. In SFT, our data suggest that this reduction in STK4 expression is driven, directly or indirectly, by the fusion protein itself, which could lead to decreased inhibitory phosphorylation of YAP1 (S127) and increased nuclear accumulation of active YAP1. This is accompanied by elevated expression of canonical Hippo target genes (*CTGF*, *ANKRD1*, *CYR61*), confirming Hippo pathway inactivation. To fully elucidate the underlying mechanism, protein–DNA binding assays would be necessary to determine whether *STK4* is directly regulated by the GF, or whether the observed reduction in STK4 mRNA and protein levels results from broader transcriptional or post-transcriptional effects of fusion expression.

Given the dysregulation of the Hippo pathway observed in our SFT model, we investigated its functional relevance by targeting YAP1/TAZ activity genetically. In this context, functional assays using siRNAs further revealed cooperation between *YAP1/TAZ* and *EGR1*. Combined knockdown of *YAP1/TAZ* and *EGR1* produced a greater reduction of *EGR1* expression than *EGR1* silencing alone, and led to decreased proliferation and reduced spheroid formation. These data are consistent with prior reports implicating EGR1 in SFT cell survival and malignancy [15], but extend them by uncovering a previously unrecognized Hippo–EGR1 functional axis. This mechanism parallels findings in other sarcomas, where YAP1 activation enhances proliferation, migration, and tumour progression [59–61]. However, the modest impact of Hippo suppression on migration in our model suggests that additional pathways may contribute to the pro-migratory phenotype of SFT-MSC.

Supporting these mechanistic insights, we explored the effect of pharmacological inhibition using dasatinib, a multi-kinase inhibitor previously reported to modulate YAP1/TAZ activity in other cancer models [45–47]. Recent studies have also demonstrated the efficacy of dasatinib in suppressing cell proliferation and malignant phenotypes in in vitro models of SFT. *Ghanim et al.* generated two patient-derived SFT pleural cell lines and showed that dasatinib exerted strong anti-proliferative effects, further enhanced in combination with trabectedin [27]. *Lee et al.* established and characterized a novel patient-derived SFT hemangiopericytoma (SFT/HPC) cell line and revealed activation of several oncogenic pathways including Epithelial–Mesenchymal Transition (EMT). Screening of 14 targeted agents revealed that dasatinib exhibited the most potent anti-tumour activity therapy [25]. Considering the well-established link between Hippo signalling and EMT [62, 63], the Hippo pathway dysregulation observed in our SFT model is consistent with the EMT activation reported in the previous study. Notably, although earlier reports have shown that SFT cells are sensitive to dasatinib, our work is, to the best of our knowledge, the first to mechanistically associate this sensitivity with Hippo pathway inactivation. Interestingly, our data show that YAP1/TAZ abrogation has a stronger impact on proliferation in 3D spheroids than in 2D cultures. This phenomenon is widely reported and underscores the context-dependent function of YAP/TAZ as mechanotransducers. In 3D environments, where cells experience spatial confinement and rely on cell–cell interactions, YAP/TAZ activity is crucial for maintaining proliferative and pro-survival programs. Consequently, loss of YAP1/TAZ may impair cell cycle progression and increase susceptibility to anoikis, contributing to the more pronounced growth reduction observed in 3D cultures [62, 64, 65]. Despite encouraging preclinical results, *Schuetze et al.* (2017) reported limited efficacy of dasatinib in a Phase 2 trial involving patients with advanced SFT, with a 6-month progression-free survival of only 30% [66]. However, given the heterogeneity of Hippo pathway activation observed in our cohort and its pronounced deregulation in recurrent or metastatic cases, dasatinib treatment in selected patients warrants further investigation. In this context, assessing Hippo pathway activity through biomarkers such as YAP1/TAZ by IHC could provide valuable predictive insights to refine patient selection and improve therapeutic outcomes in future clinical trials [67].

At the clinical level, and consistent with previous reports [23, 54], our RNA-seq analysis of patient tumour samples revealed that SFT transcriptomic profiles vary according to tumour location. Notably, ssGSEA scores showed a positive trend between Cordenonsi YAP Conserved and EGR1 signatures although the correlation did not reach statistical significance. In addition, *YAP1* expression closely correlated with several established EGR1 targets (*NAB1*, *IGF2*, *FGF2* and *FGFR1*), whereas *WWTR1* showed no significant association with these targets, despite being linked to *EGR1* expression and the Hippo signalling pathway targets (*CYR61* and *CTGF*). This point is particularly interesting because most studies agree that YAP1 activity is primarily regulated at the protein level. Key mechanisms, such as phosphorylation by LATS kinases, nuclear–cytoplasmic shuttling, and interactions with TEAD, control its transcriptional output far more effectively than changes in mRNA abundance [46, 58]. Accordingly, our results are consistent with these observations: *WWTR1* mRNA levels, but not *YAP1*, correlate with the expression of Hippo target genes. However, we observed correlation amongst *YAP1* and EGR1 target genes, suggesting that correlations of *YAP1* at mRNA levels may also reflect its transcriptional activity in other contexts. This pattern proposes that YAP1 and EGR1 may cooperate, possibly through a protein–protein interaction, to regulate EGR1’s downstream genes. Indeed, previous studies have demonstrated that EGR1 can physically bind to YAP1 via its WW domain, forming a complex that enhances EGR1-driven transcriptional activity in prostate carcinoma cells [68]. Moreover, YAP/TAZ have been described as broadly required for the induction of immediate early genes, including *EGR1* [69]. In the context of SFTs—where EGR1´s role is especially critical due to its association with the NAB2::STAT6 fusion protein, our results suggest that YAP1 may become essential in cooperating with EGR1 to regulate the oncogenic program of the SFT cells.

Furthermore, YAP1/TAZ IHC demonstrated heterogeneous nuclear staining among patients, consistent with previous analyses reporting moderate to strong nuclear staining of YAP1 in 42% (15/36) of SFT evaluated [70]. Notably, higher nuclear YAP1/TAZ expression correlated with patient age and showed a marked trend toward association with extrapleural tumour location. Importantly, extrathoracic tumour location has been identified as an independent predictor of poor prognosis [71] and patient age ≥ 55 years is a recognized adverse prognostic factor in SFT, incorporated into the *Demicco* risk stratification model [7]. In line with these observations, relapsed tumours, particularly second relapses, exhibited stronger nuclear YAP1/TAZ staining than their matched primary counterparts, further supporting the hypothesis that inactivation of the Hippo pathway may contribute to tumour aggressiveness and therapy resistance. Nevertheless, no significant association with patient outcome was detected, which may be attributable to the limited follow-up period (median 4–6 years) of our series and the characteristically late-recurring nature of SFT, as approximately one-third of recurrences occur beyond five years and patients remain at risk even a decade after surgery [72]. These findings underscore the need for long-term clinical follow-up and larger, well-characterized patient cohorts to more accurately determine the prognostic significance of YAP1/TAZ expression and other emerging molecular biomarkers in SFT.

OGM analysis revealed few large SVs but numerous small, focal rearrangements, underscoring the overall genomic simplicity of SFTs. This simplicity extends to core Hippo pathway genes, in which no structural alterations were detected, suggesting that Hippo pathway dysregulation in SFTs arises primarily at the transcriptional or post-transcriptional level rather than through genomic events. This observation aligns with early integrative sequencing studies, which showed that SFTs are largely defined by the characteristic NAB2::STAT6 GF and display minimal additional genomic alterations [15, 73]. Interestingly, SFT_7 exhibited several STAT6-related SVs, also confirmed at the mRNA by total RNA-seq, suggesting that the NAB2::STAT6 fusion may have arisen from a complex rearrangement rather than a simple inversion. The clinical implications of this complex SV warrant further investigation, as tumours—including sarcomas—with complex genomic rearrangements (such as chromoplexy) have been associated with poorer prognosis [74]. Consistently, this patient, despite being classified as “low risk” by the *Demicco* model [7], developed pulmonary metastasis more than 10 years after the initial diagnosis. Expanding OGM analyses to larger cohorts will be essential to clarify the prognostic relevance of complex SVs. However, the rarity of SFT and the requirement for high-quality frozen tissue remain significant limiting factors.

In summary, our study establishes a novel mechanistic framework in which *NAB2::STAT6* promotes SFT aggressiveness, through deregulation of the Hippo pathway activity, leading to YAP1/TAZ activation and cooperation with EGR1-driven programs (Supplementary Fig. 6). These findings indicate that Hippo signalling plays an important role in SFT biology and may constitute a therapeutic vulnerability. Although our data are consistent with in vitro sensitivity to dasatinib, these observations remain exploratory and further studies will be required to establish the therapeutic relevance of these observations. Despite the robustness of our multi-level approach, certain inherent limitations must be acknowledged: the rarity of SFT constrained the size of the patient cohort, and the relatively short follow-up period may have reduced the statistical power of our analyses. Moreover, while our mesenchymal model recapitulates key molecular and functional features of SFT, it lacks the complexity of the tumour microenvironment. Future studies using PDX or organoid systems will be essential to validate the NAB2::STAT6–Hippo–EGR1 axis and to evaluate combinatorial strategies involving YAP/TAZ inhibitors such as dasatinib.

## Supplementary Information

Below is the link to the electronic supplementary material.

Supplementary Material 1: Figure 1. Characterization of SFT-MSC models expressing NAB2::STAT6. A) Relative STAT6 mRNA levels in c-MSC (control) and SFT-MSC measured by qPCR. GAPDH was used as loading control. Data represent mean ± SD (n = 3). B) Western blot analysis of STAT6 protein expression via HA epitope. GAPDH served as loading control. Representative images from three independent experiments are shown. C) Heatmap of unsupervised hierarchical clustering of c-MSC (red) and SFT-MSC (blue) samples showing transcriptional profiles. D) t-SNE2 plot showing the segregation of c-MSC and SFT-MSC models alongside clinical SFT samples. E) Hierarchical clustering of c-MSC and SFT-MSC models with clinical SFT samples based on their transcriptomic profiles. DNA alteration reported in the Hajdu M. et al [31] study: CA_SFT10_HG-U133A and CA_SFT38_HG-U133A: 6::17; CA_SFT18_HG-U133A: 6::18; and CA_SFT40_HG-U133A: 4::3 F) Flow cytometry analysis of Cherry reporter expression in wt-MSC (grey) versus SFT-MSC #1 and SFT-MSC #2 (red). Representative from ≥3 experiments. G) Western blot analysis of STAT6 protein levels in nuclear and cytoplasmic fractions. α-TUBULIN (cytoplasm) and Histone H3 (nucleus) used as loading controls. H) Flow cytometry scatter plots showing changes in cell size (FSC) and complexity (SSC) between c-MSC (grey) and SFT-MSC #1 (left) / SFT-MSC #2 (right) (red). I) Representative bright-field (left) and Calcein-AM fluorescence (right) images showing morphological changes. J) Relative FGF2 and FGFR1 mRNA levels in c-MSC and SFT-MSC assessed by qPCR. GAPDH was used as loading control. Data represent mean ± SD (n = 3).

Supplementary Material 2: Figure 2. YAP/TAZ knockdown silences targets without affecting SFT-MSC migration. A) qPCR analysis of Hippo pathway genes after 24 h siRNA treatment. GAPDH was used as a loading control; c-MSC served as the reference condition. GAPDH was used as loading control. Data represent mean ± SD (n = 3). B) Representative images of wound healing assays after siRNA treatment, shown at baseline 0- and 6-hours post-wound induction.

Supplementary Material 3: Figure 3. ssGSEA enrichment of YAP conserved and EGR1 signatures in SFT samples. ssGSEA plots for SFT tumor samples (SFT_5, SFT_10, SFT_1) showing enrichment of the YAP conserved signature (blue) and EGR1_01 signature (orange). Genes are ranked by expression, and normalized enrichment scores (NES) indicate pathway activation in each sample.

Supplementary Material 4: Figure 4. Clinical correlation with nuclear YAP1/TAZ staining. A) Summary table of clinical associations in the SFT patient cohort, stratified into Low and High groups based on the median nuclear YAP1/TAZ staining. The table lists clinical variables, the number and percentage of cases in each group, and corresponding p-value from the statistical analysis. Means are showed with their SD. B) YAP1/TAZ total staining score (TSS) represented as continuous variable according to patient age (<55 vs. ≥55 years) (left) and tumour location (right) in primary tumours. C) YAP1/TAZ TSS represented as continuous variable according to tumour tissue (primary or recidive).

Supplementary Material 5: Figure 5. Comprehensive analysis of structural variants in SFT samples using Optical Genome Mapping (OGM). A) Copy number variation (CNV) profile across seven SFT patient samples. Gains are shown in blue; and losses in red. B) Circos plots illustrating the structural variants (SVs) identified in each SFT case by OGM. The legend specifies the number and type of SVs, each represented by a distinct color. C) Visualization of the NAB2::STAT6 structural variant identified as a deletion by OGM. The aberrant inversion in the 12q13–15 region is interpreted as a deletion event. D) Detection of a large interchromosomal SV involving chromosomes 4 and 12 in SFT case #7.

Supplementary Material 6: Figure 6. Schematic model of Hippo pathway deregulation in mesenchymal cells expressing NAB2::STAT6. Expression of the NAB2::STAT6 fusion protein drives transcription of EGR1 target genes, consistent with the model proposed by Robinson et al. [15]. This is accompanied by reduced expression and activating phosphorylation of STK4/MST1, though the precise mechanism of MST1 repression remains unclear (?). As a result, decreased phosphorylation of downstream co-factors YAP1/TAZ allows their nuclear translocation and activation of target genes that promote cell growth and survival.

Supplementary Material 7

Supplementary Material 8: Table 1_Material and methods

Supplementary Material 9: Table 2_Gene Expression Assay

Supplementary Material 10: Table 3_SFT patient cohort

Supplementary Material 11: Table 4_ Gene fusions by RNAseq

Supplementary Material 12: Table 5_OGM quality parameters

Supplementary Material 13: Table 6_SVs by OGM

## Data Availability

No datasets were generated or analysed during the current study.
