# Supplementary figures and images for: Hippo signalling pathway mediates oncogenic properties of NAB2::STAT6 in solitary fibrous tumour
Source: Cell Oncol (Dordr). 2026 Feb 10;49(1):43. doi: 10.1007/s13402-026-01173-x (PMC12891060; doi:10.1007/s13402-026-01173-x)

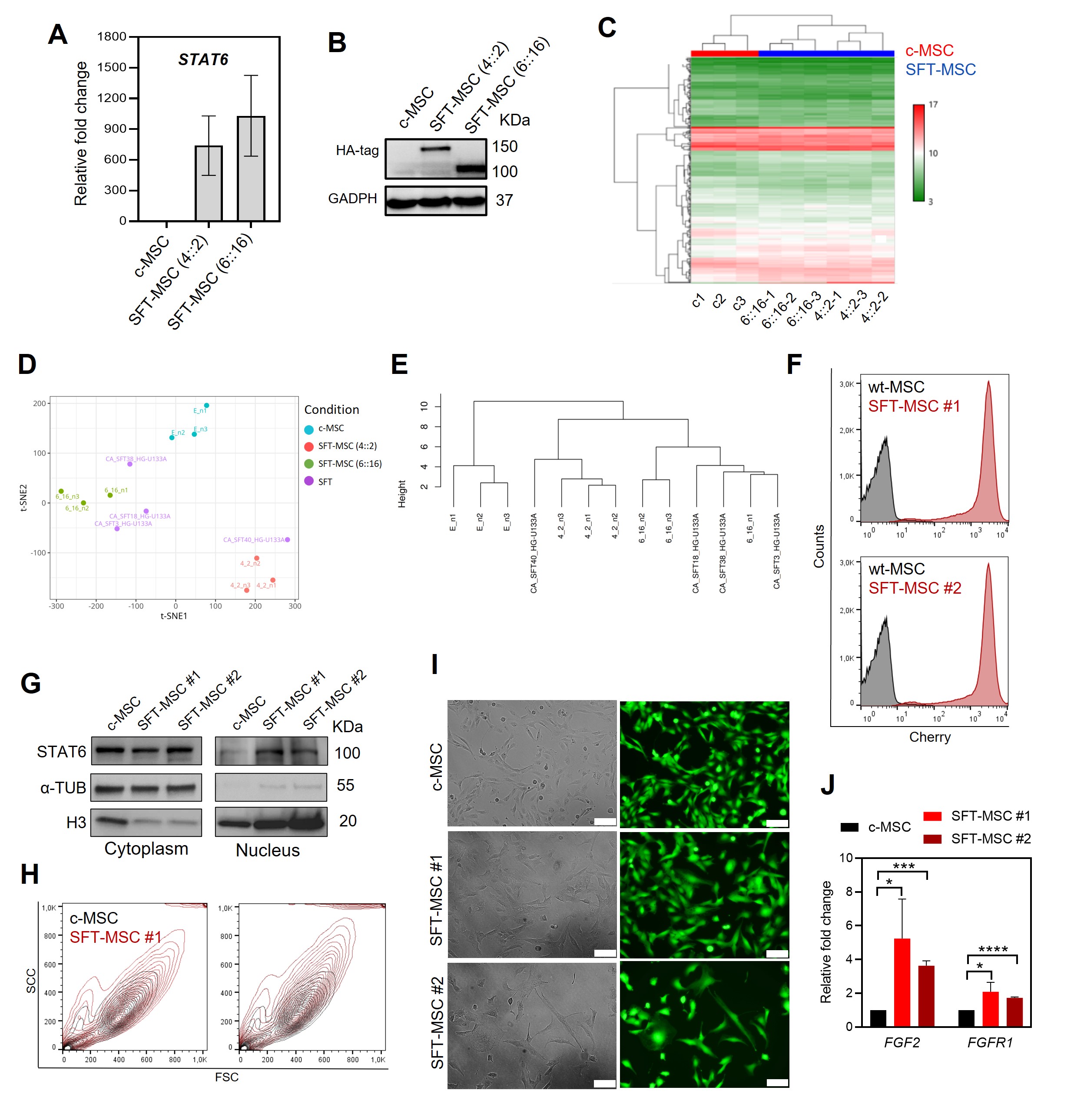

Supplement: Supplementary file 1 — Supplementary Material 1: Figure 1. Characterization of SFT-MSC models expressing NAB2::STAT6. A) Relative STAT6 mRNA levels in c-MSC (control) and SFT-MSC measured by qPCR. GAPDH was used as loading control. Data represent mean ± SD (n = 3). B) Western blot analysis of STAT6 protein expression via HA epitope. GAPDH served as loading control. Representative images from three independent experiments are shown. C) Heatmap of unsupervised hierarchical clustering of c-MSC (red) and SFT-MSC (blue) samples showing transcriptional profiles. D) t-SNE2 plot showing the segregation of c-MSC and SFT-MSC models alongside clinical SFT samples. E) Hierarchical clustering of c-MSC and SFT-MSC models with clinical SFT samples based on their transcriptomic profiles. DNA alteration reported in the Hajdu M. et al [31] study: CA_SFT10_HG-U133A and CA_SFT38_HG-U133A: 6::17; CA_SFT18_HG-U133A: 6::18; and CA_SFT40_HG-U133A: 4::3 F) Flow cytometry analysis of Cherry reporter expression in wt-MSC (grey) versus SFT-MSC #1 and SFT-MSC #2 (red). Representative from ≥3 experiments. G) Western blot analysis of STAT6 protein levels in nuclear and cytoplasmic fractions. α-TUBULIN (cytoplasm) and Histone H3 (nucleus) used as loading controls. H) Flow cytometry scatter plots showing changes in cell size (FSC) and complexity (SSC) between c-MSC (grey) and SFT-MSC #1 (left) / SFT-MSC #2 (right) (red). I) Representative bright-field (left) and Calcein-AM fluorescence (right) images showing morphological changes. J) Relative FGF2 and FGFR1 mRNA levels in c-MSC and SFT-MSC assessed by qPCR. GAPDH was used as loading control. Data represent mean ± SD (n = 3). [file 13402_2026_1173_MOESM1_ESM.jpg]

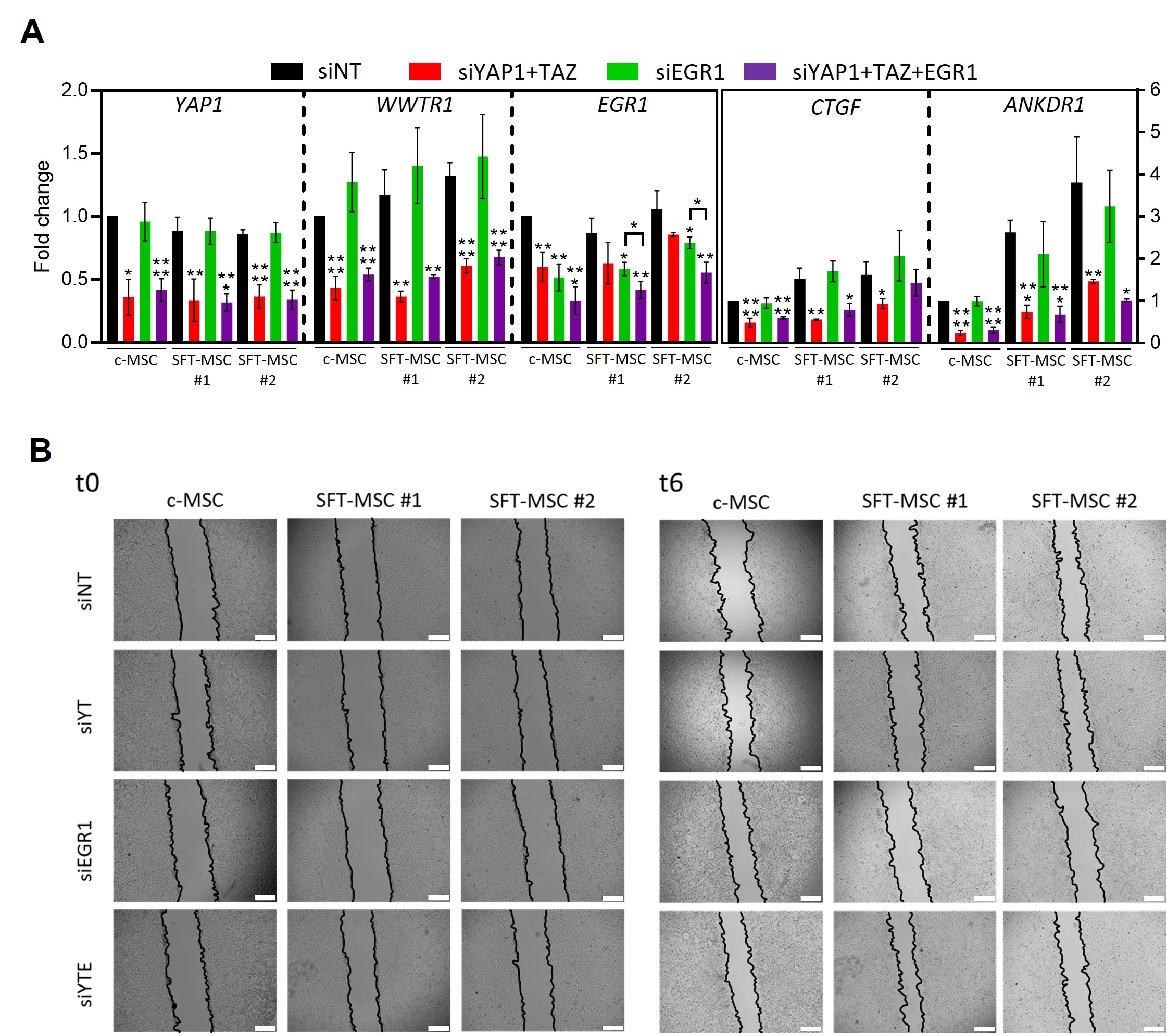

Supplement: Supplementary file 2 — Supplementary Material 2: Figure 2. YAP/TAZ knockdown silences targets without affecting SFT-MSC migration. A) qPCR analysis of Hippo pathway genes after 24 h siRNA treatment. GAPDH was used as a loading control; c-MSC served as the reference condition. GAPDH was used as loading control. Data represent mean ± SD (n = 3). B) Representative images of wound healing assays after siRNA treatment, shown at baseline 0- and 6-hours post-wound induction. [file 13402_2026_1173_MOESM2_ESM.jpg]

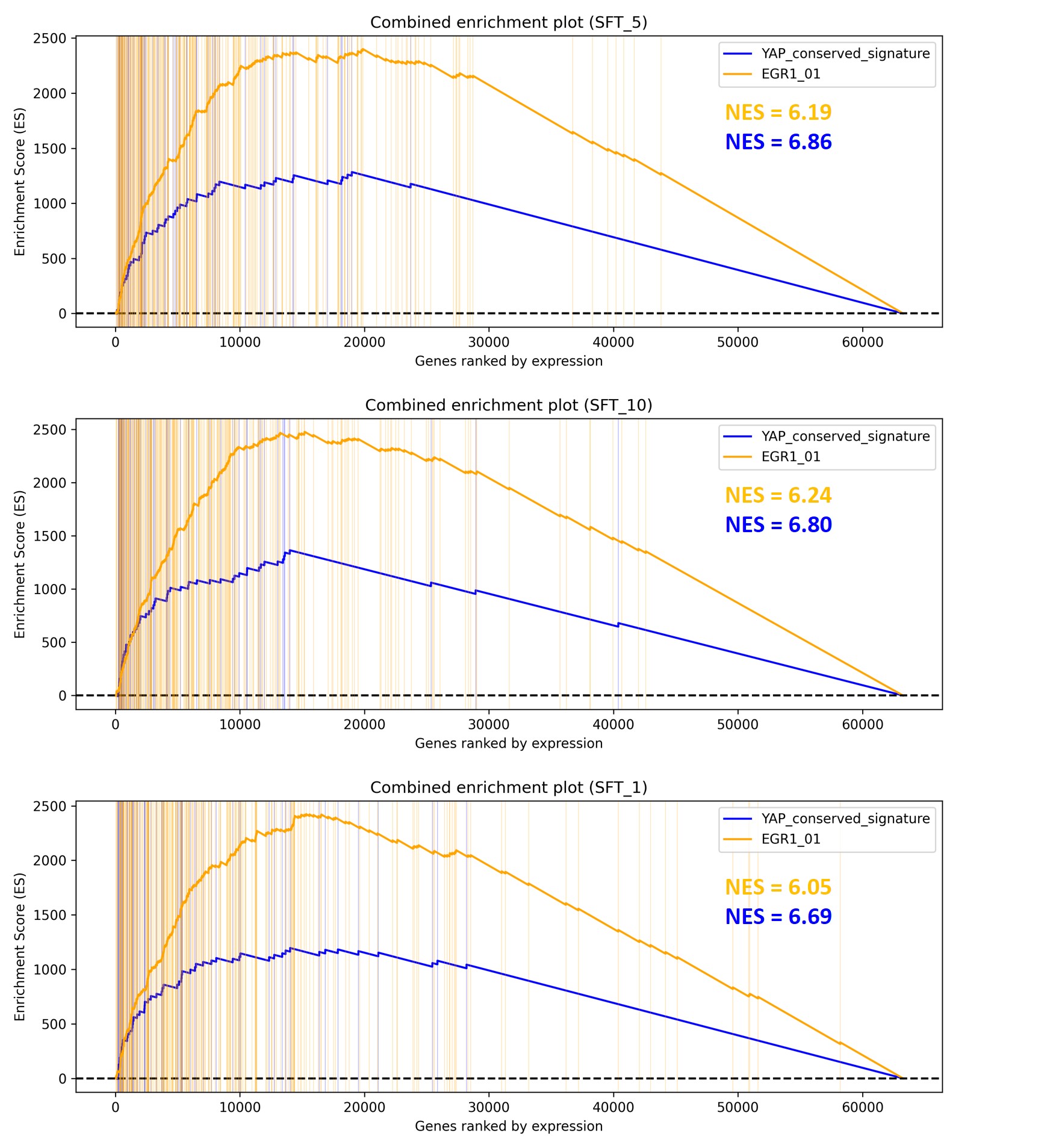

Supplement: Supplementary file 3 — Supplementary Material 3: Figure 3. ssGSEA enrichment of YAP conserved and EGR1 signatures in SFT samples. ssGSEA plots for SFT tumor samples (SFT_5, SFT_10, SFT_1) showing enrichment of the YAP conserved signature (blue) and EGR1_01 signature (orange). Genes are ranked by expression, and normalized enrichment scores (NES) indicate pathway activation in each sample. [file 13402_2026_1173_MOESM3_ESM.jpg]

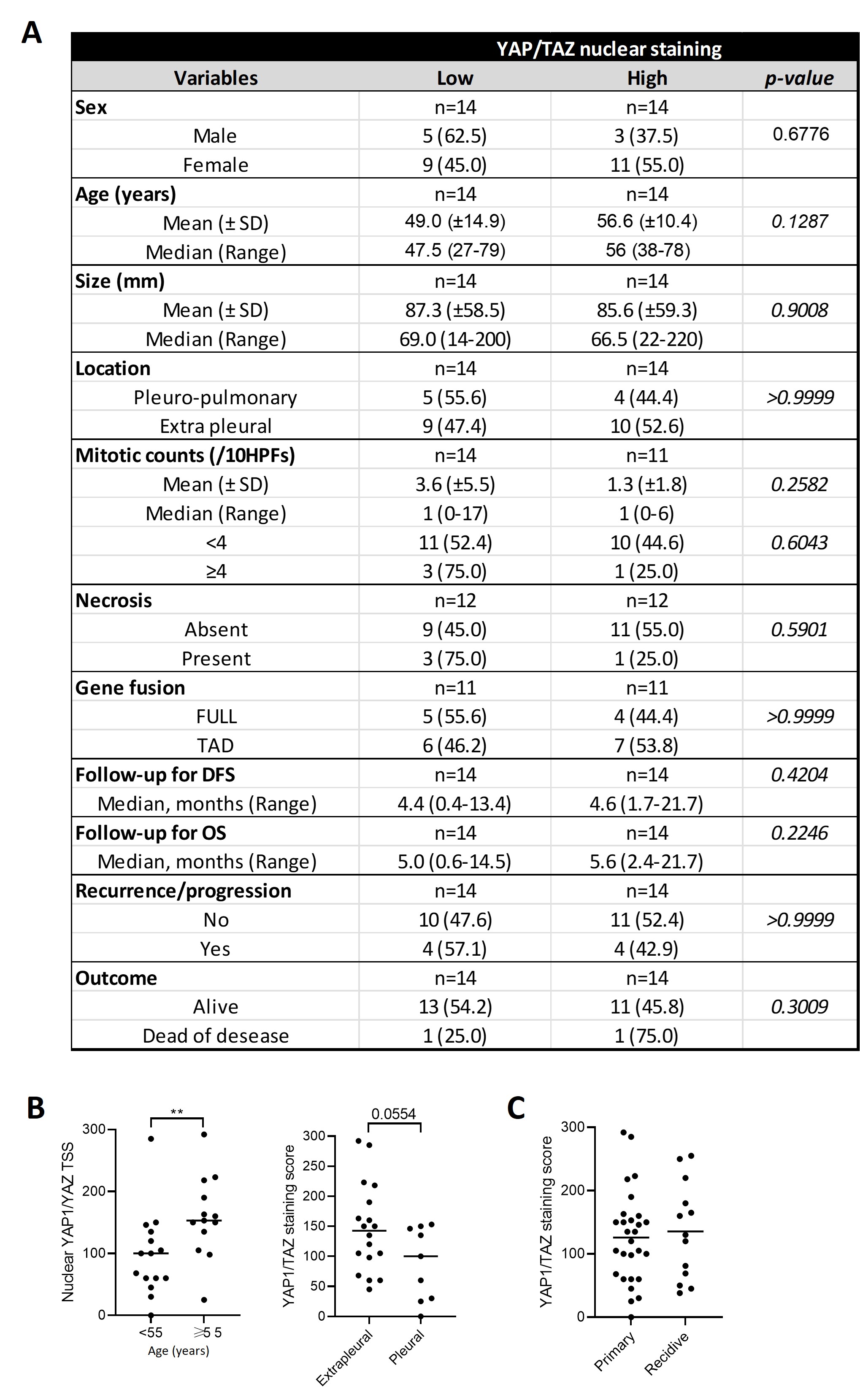

Supplement: Supplementary file 4 — Supplementary Material 4: Figure 4. Clinical correlation with nuclear YAP1/TAZ staining. A) Summary table of clinical associations in the SFT patient cohort, stratified into Low and High groups based on the median nuclear YAP1/TAZ staining. The table lists clinical variables, the number and percentage of cases in each group, and corresponding p-value from the statistical analysis. Means are showed with their SD. B) YAP1/TAZ total staining score (TSS) represented as continuous variable according to patient age (<55 vs. ≥55 years) (left) and tumour location (right) in primary tumours. C) YAP1/TAZ TSS represented as continuous variable according to tumour tissue (primary or recidive). [file 13402_2026_1173_MOESM4_ESM.jpg]

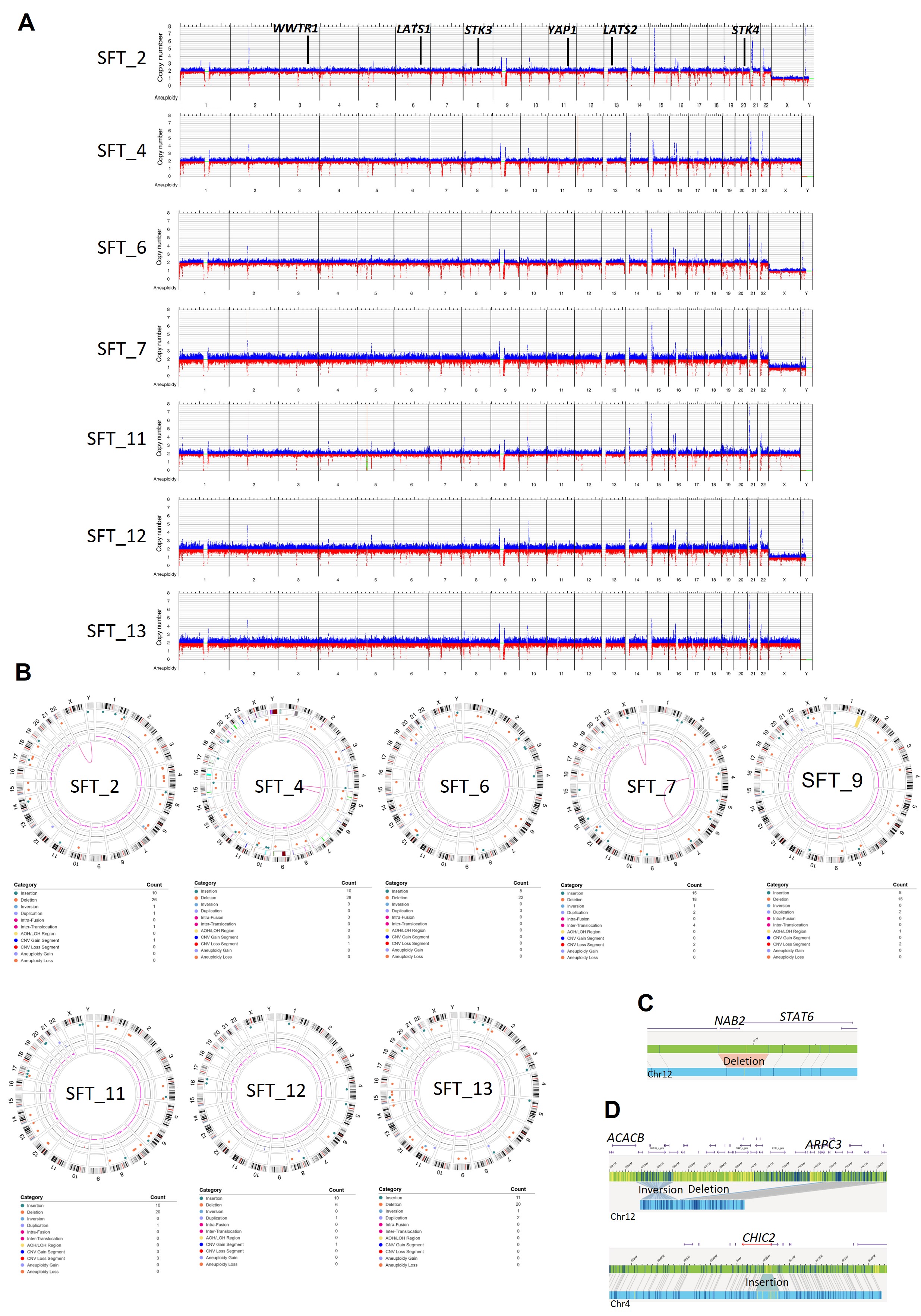

Supplement: Supplementary file 5 — Supplementary Material 5: Figure 5. Comprehensive analysis of structural variants in SFT samples using Optical Genome Mapping (OGM). A) Copy number variation (CNV) profile across seven SFT patient samples. Gains are shown in blue; and losses in red. B) Circos plots illustrating the structural variants (SVs) identified in each SFT case by OGM. The legend specifies the number and type of SVs, each represented by a distinct color. C) Visualization of the NAB2::STAT6 structural variant identified as a deletion by OGM. The aberrant inversion in the 12q13–15 region is interpreted as a deletion event. D) Detection of a large interchromosomal SV involving chromosomes 4 and 12 in SFT case #7. [file 13402_2026_1173_MOESM5_ESM.jpg]

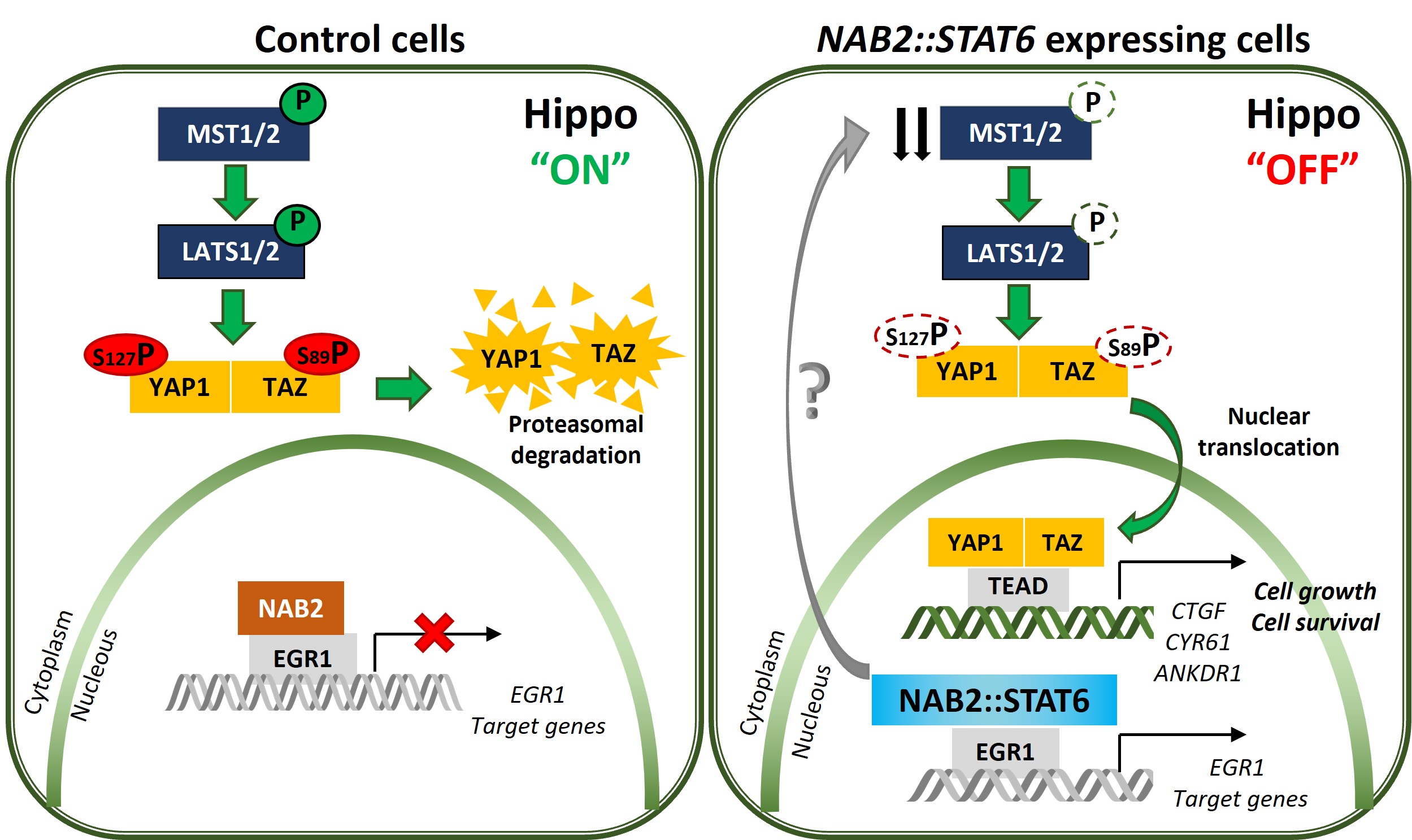

Supplement: Supplementary file 6 — Supplementary Material 6: Figure 6. Schematic model of Hippo pathway deregulation in mesenchymal cells expressing NAB2::STAT6. Expression of the NAB2::STAT6 fusion protein drives transcription of EGR1 target genes, consistent with the model proposed by Robinson et al. [15]. This is accompanied by reduced expression and activating phosphorylation of STK4/MST1, though the precise mechanism of MST1 repression remains unclear (?). As a result, decreased phosphorylation of downstream co-factors YAP1/TAZ allows their nuclear translocation and activation of target genes that promote cell growth and survival. [file 13402_2026_1173_MOESM6_ESM.jpg]
