# Supplementary material for: Hippo signalling pathway mediates oncogenic properties of NAB2::STAT6 in solitary fibrous tumour
Source: Cell Oncol (Dordr). 2026 Feb 10;49(1):43. doi: 10.1007/s13402-026-01173-x (PMC12891060; doi:10.1007/s13402-026-01173-x)

Figure 1E

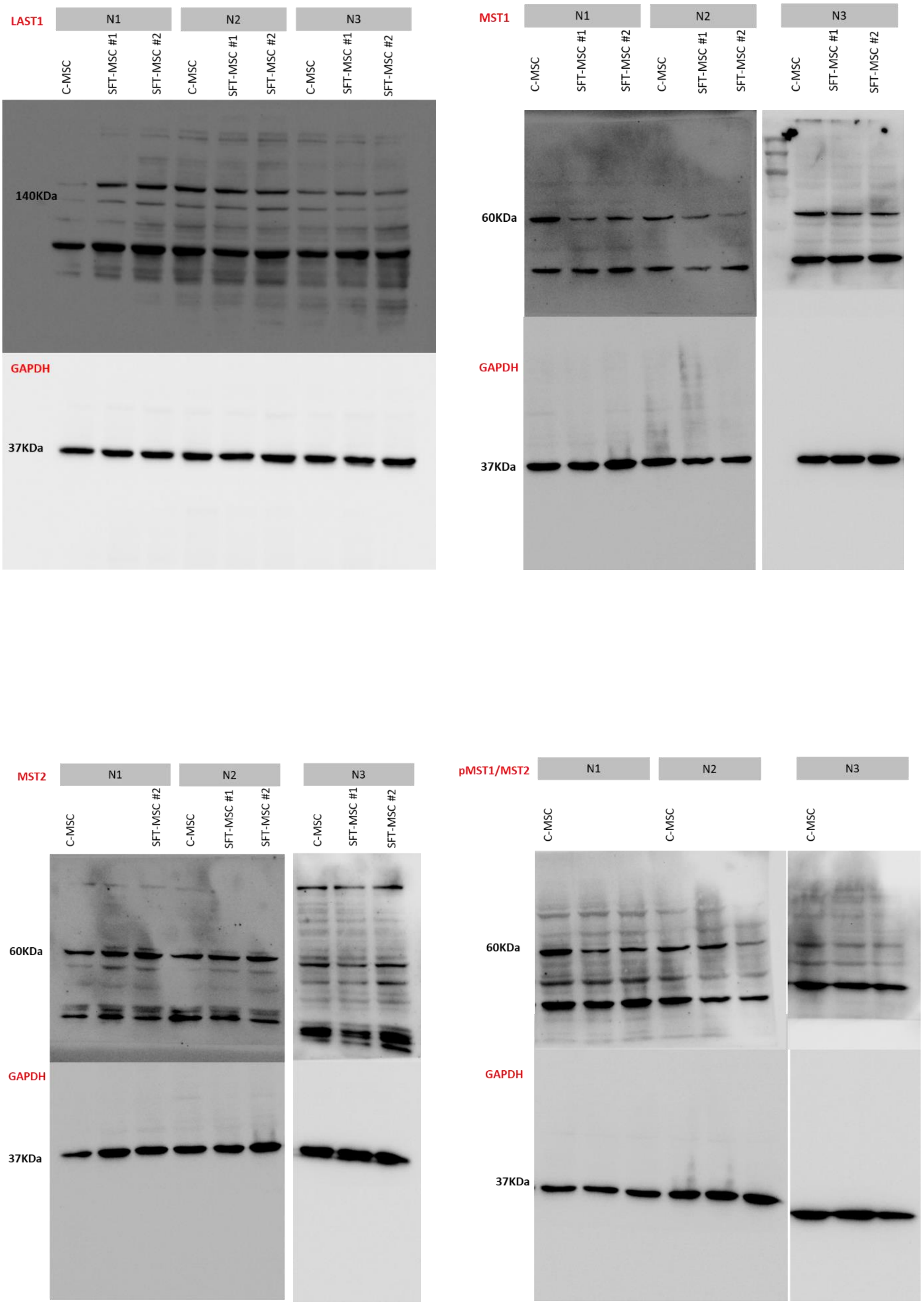
LAST1
N1
N2
N3
C-MSC
SFT-MSC #1
SFT-MSC #2
C-MSC
SFT-MSC #1
SFT-MSC #2
C-MSC
SFT-MSC #1
SFT-MSC #2
140KDa
GAPDH
37KDa
MST1
N1
N2
N3
C-MSC
SFT-MSC #1
SFT-MSC #2
C-MSC
SFT-MSC #1
SFT-MSC #2
C-MSC
SFT-MSC #1
SFT-MSC #2
60KDa
GAPDH
37KDa
MST2
N1
N2
N3
C-MSC
SFT-MSC #2
C-MSC
SFT-MSC #1
SFT-MSC #2
C-MSC
SFT-MSC #1
SFT-MSC #2
60KDa
GAPDH
37KDa
pMST1/MST2
N1
N2
N3
C-MSC
C-MSC
C-MSC
60KDa
GAPDH
37KDa

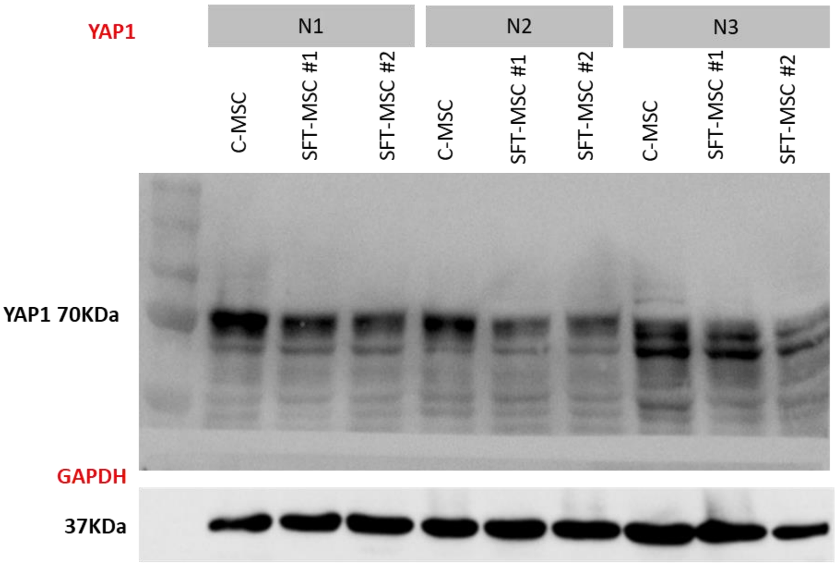
YAP1
N1
N2
N3
C-MSC
SFT-MSC #1
SFT-MSC #2
C-MSC
SFT-MSC #1
SFT-MSC #2
C-MSC
SFT-MSC #1
SFT-MSC #2
YAP1 70KDa
GAPDH
37KDa

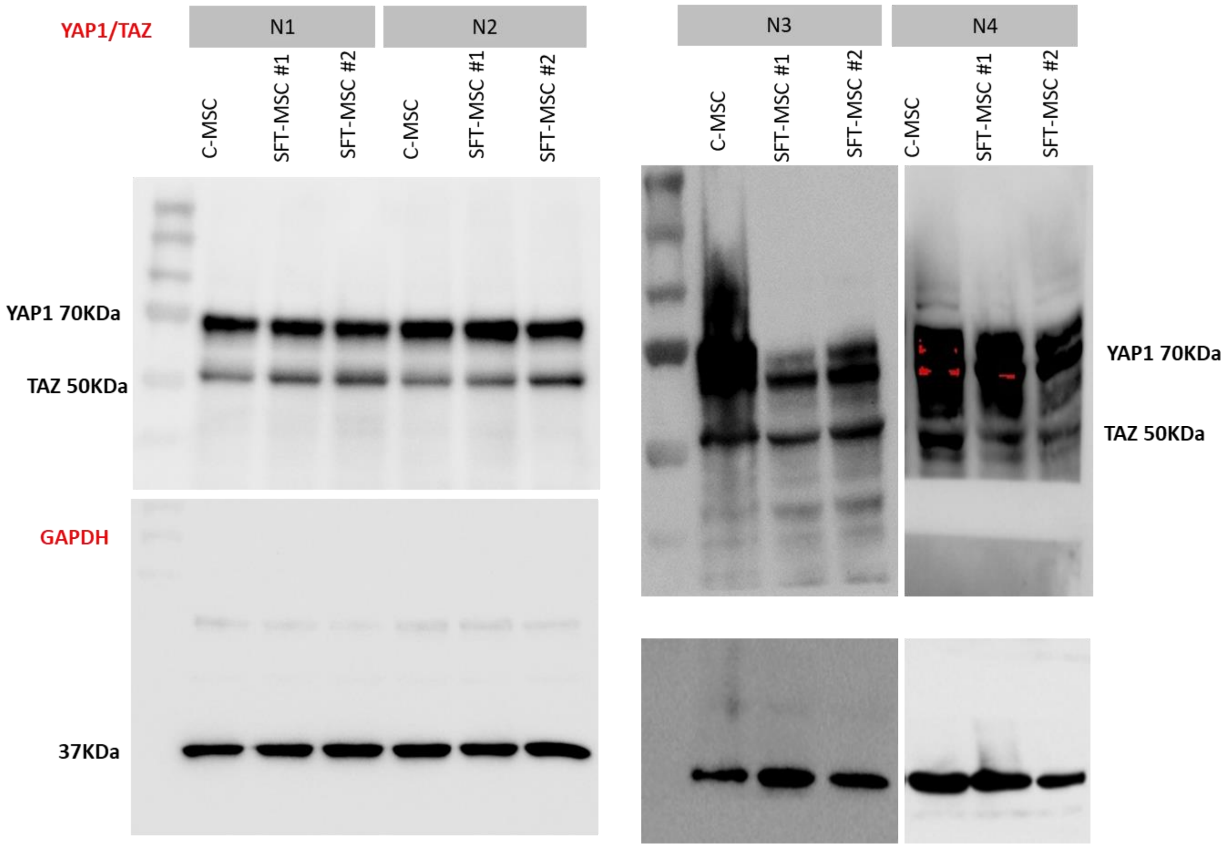
YAP1/TAZ
N1
N2
C-MSC
SFT-MSC #1
SFT-MSC #2
C-MSC
SFT-MSC #1
SFT-MSC #2
YAP1 70KDa
TAZ 50KDa
GAPDH
37KDa
N3
N4
C-MSC
SFT-MSC #1
SFT-MSC #2
C-MSC
SFT-MSC #1
SFT-MSC #2
YAP1 70KDa
TAZ 50KDa

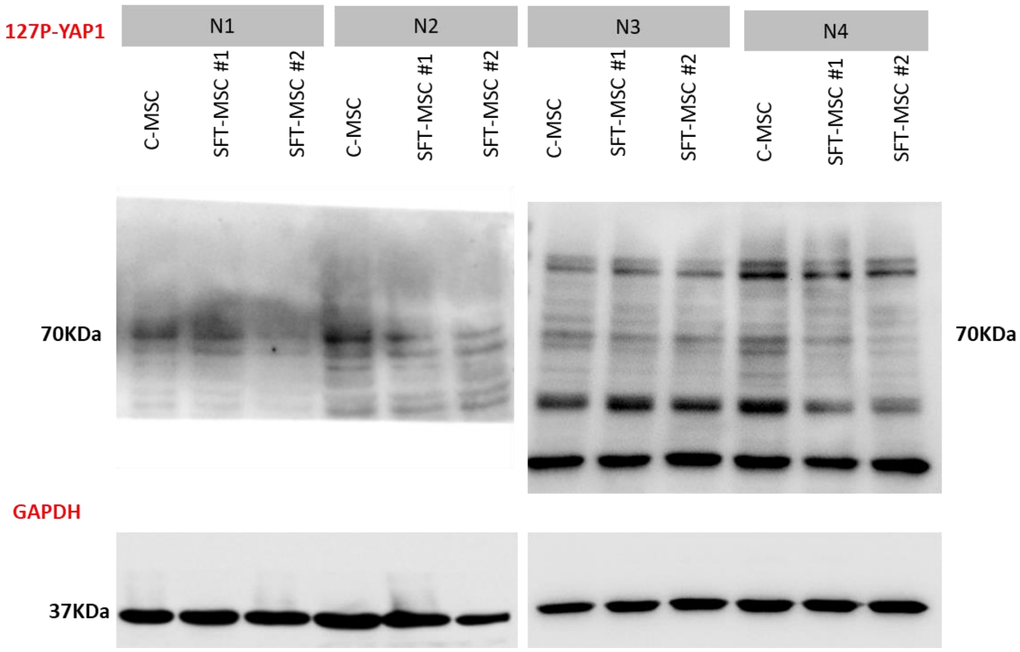
127P-YAP1
N1
N2
N3
N4
C-MSC
SFT-MSC #1
SFT-MSC #2
C-MSC
SFT-MSC #1
SFT-MSC #2
C-MSC
SFT-MSC #1
SFT-MSC #2
C-MSC
SFT-MSC #1
SFT-MSC #2
70KDa
70KDa
GAPDH
37KDa

Figure 3B

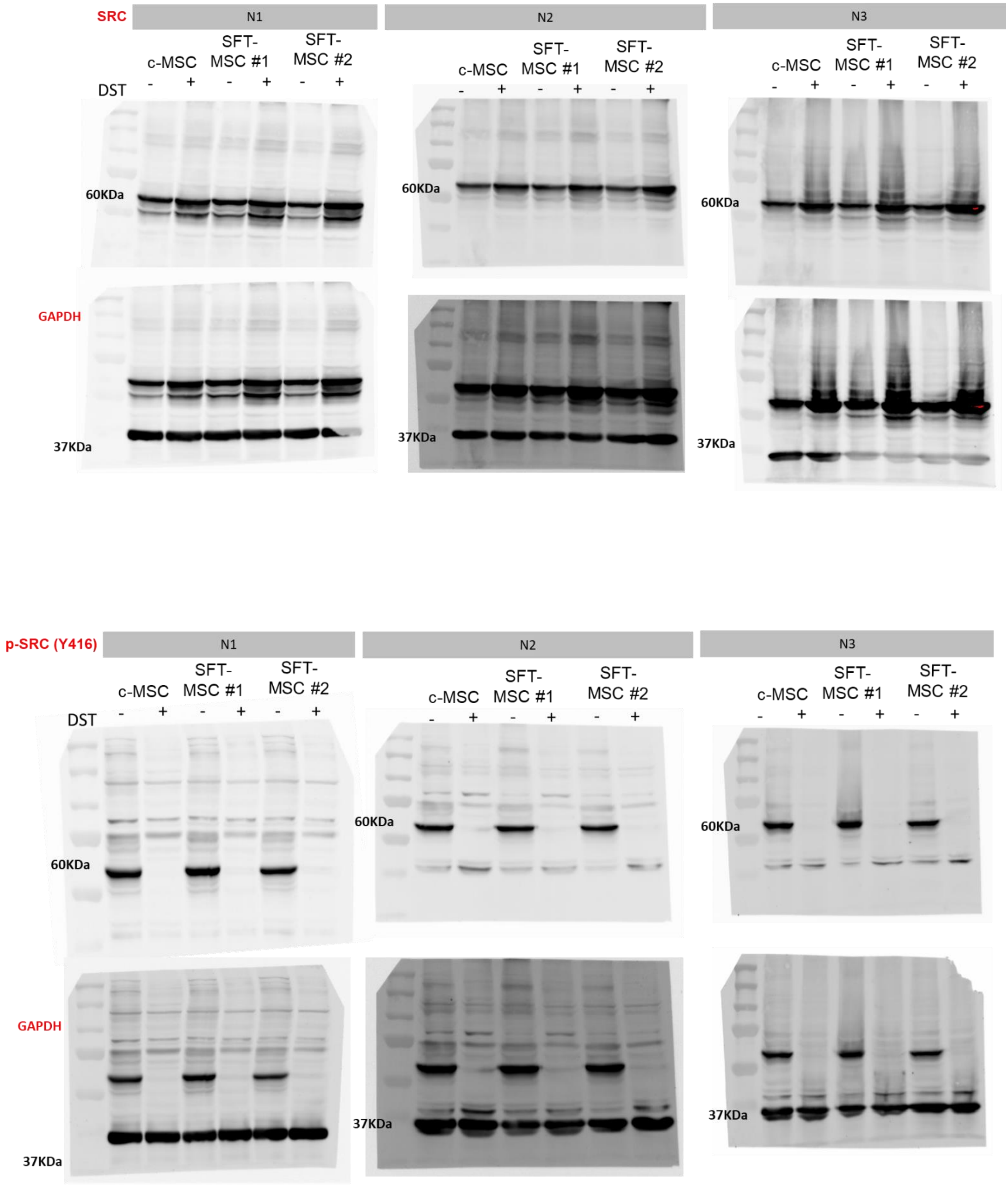
SRC
N1
N2
N3
c-MSC
SFT-MSC #1
SFT-MSC #2
DST
- + - + - +
60KDa
GAPDH
37KDa
p-SRC (Y416)
N1
N2
N3
c-MSC
SFT-MSC #1
SFT-MSC #2
DST
- + - + - +
60KDa
GAPDH
37KDa

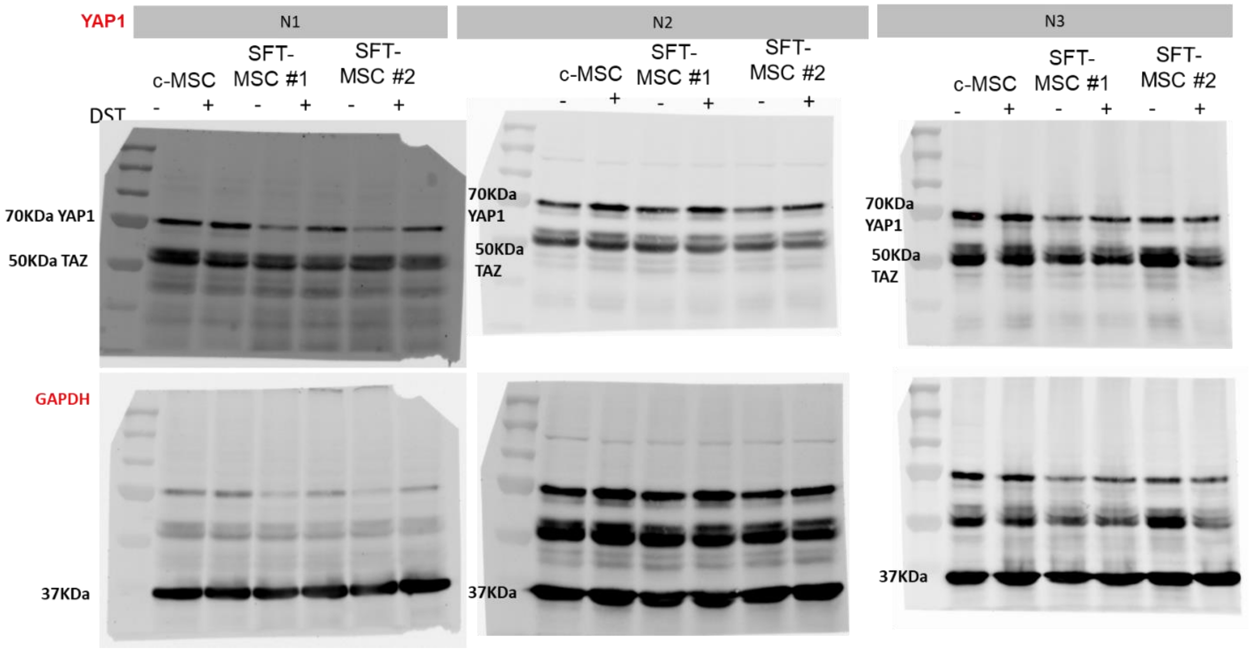
YAP1
N1
N2
N3
c-MSC
SFT-
MSC #1
SFT-
MSC #2
DST
-
+
-
+
-
+
70KDa YAP1
50KDa TAZ
70KDa
YAP1
50KDa
TAZ
GAPDH
37KDa

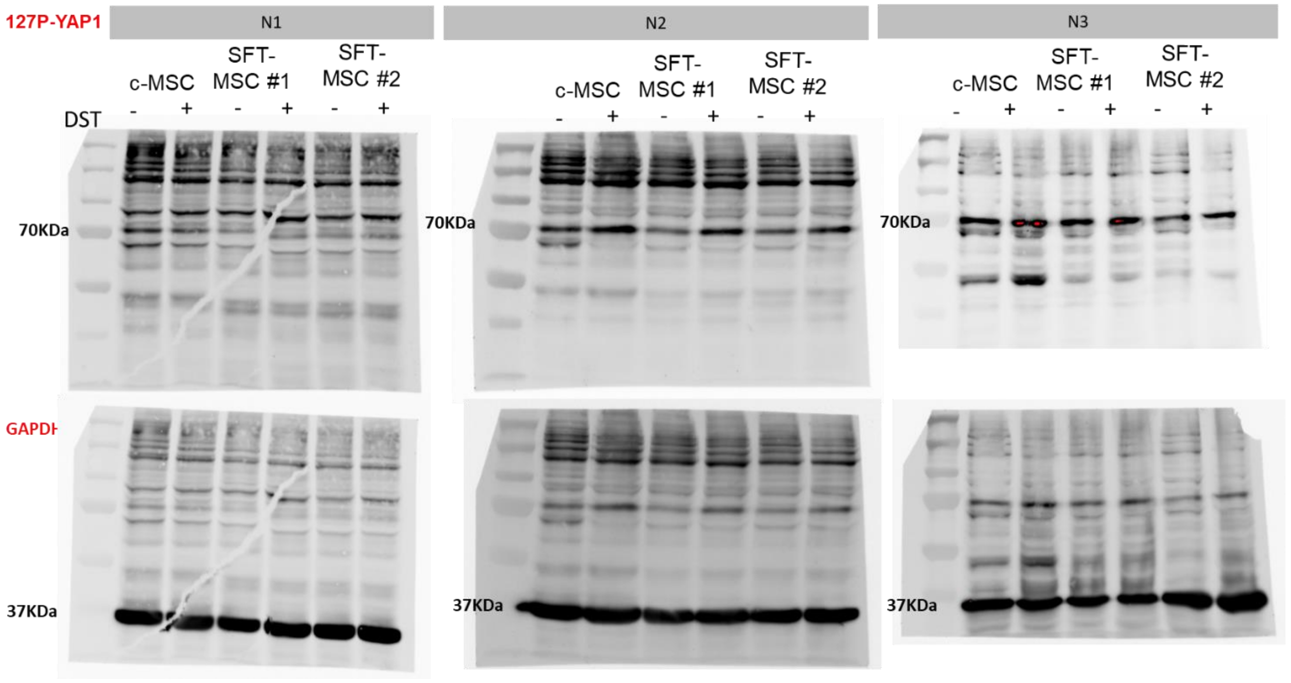
127P-YAP1
N1
N2
N3
c-MSC
SFT-
MSC #1
SFT-
MSC #2
DST
-
+
-
+
-
+
70KDa
GAPDH
37KDa

Supplementary Fig1B

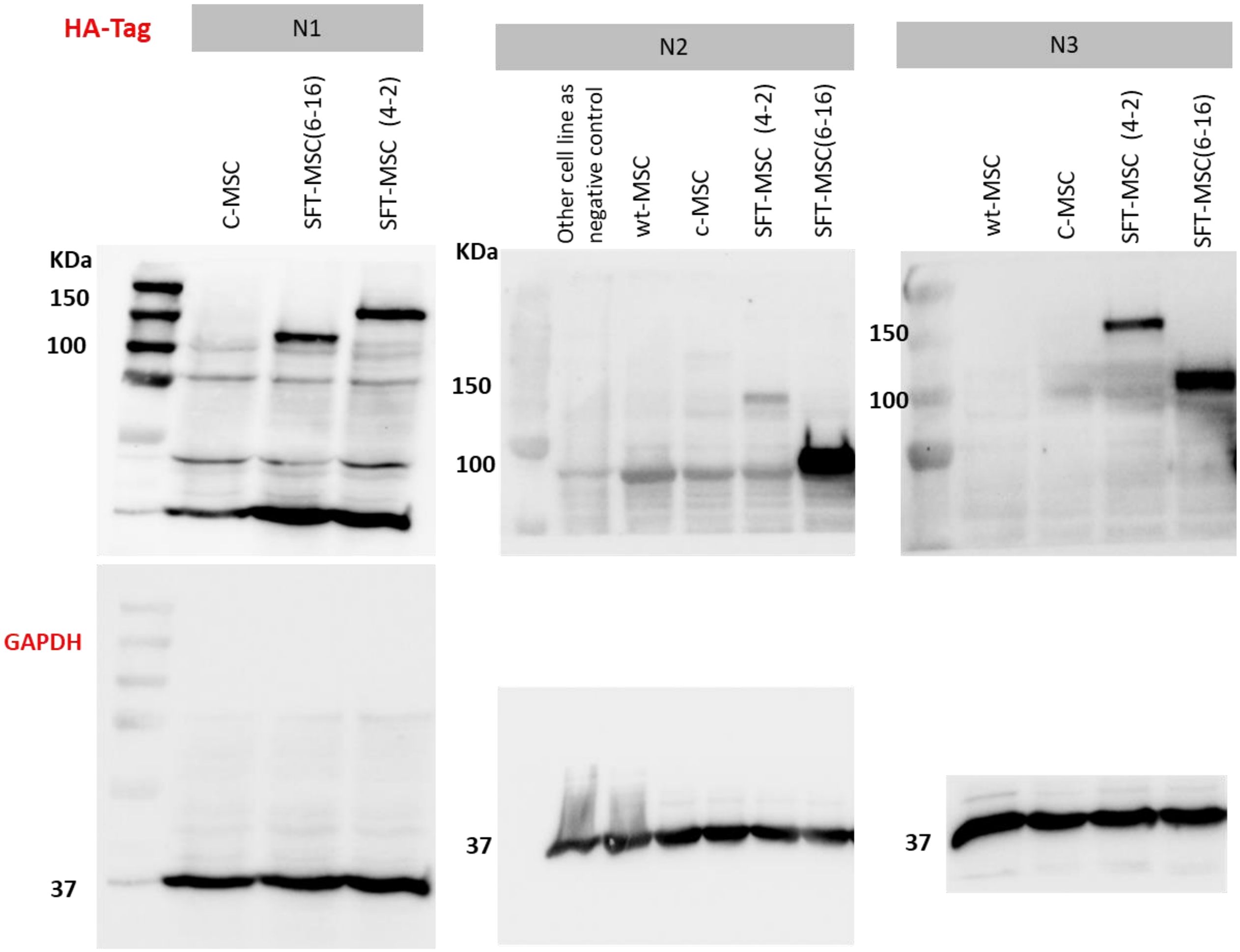

Supplementary Fig1E

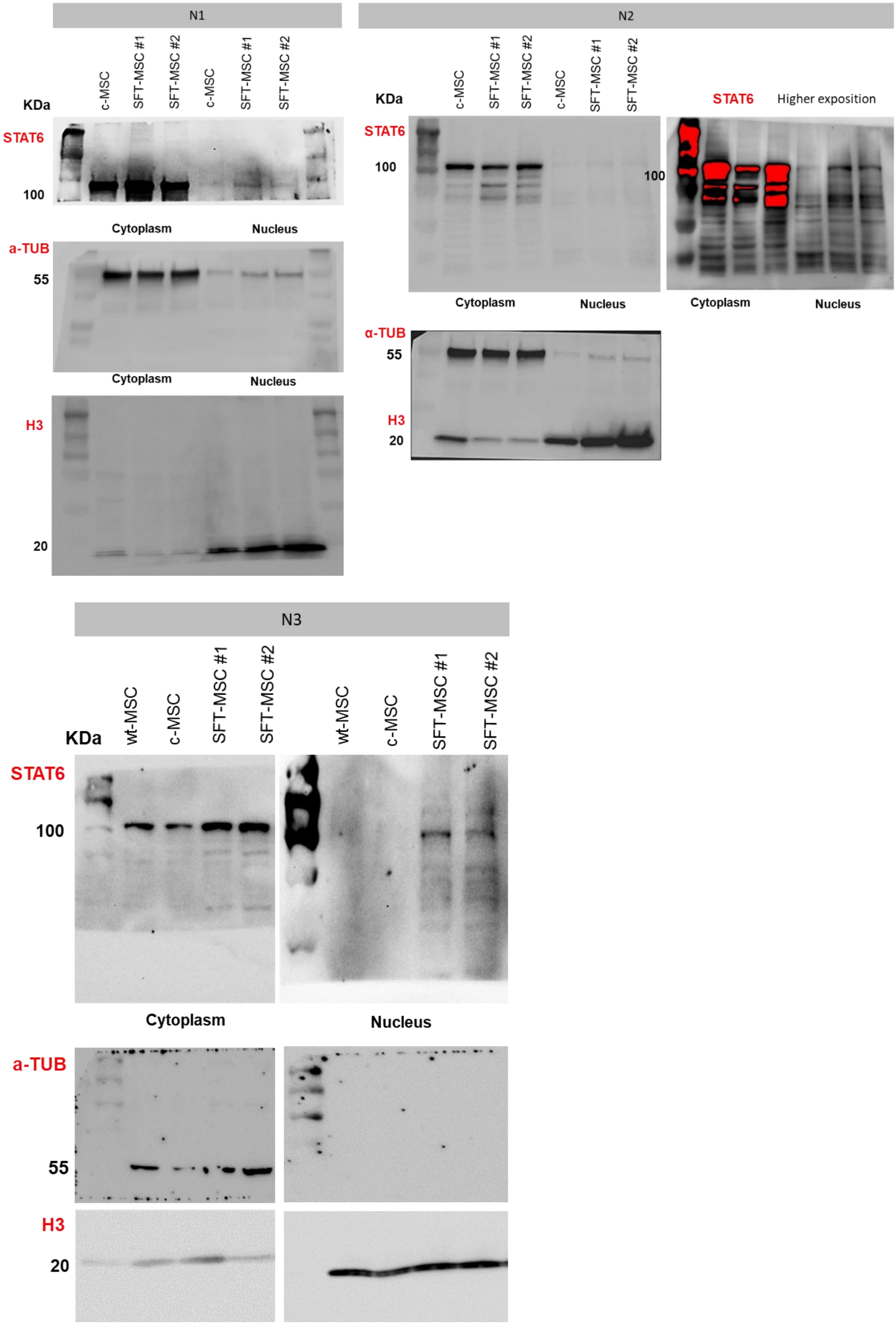

Supplement: Supplementary file 7 — Supplementary Material 7 [file 13402_2026_1173_MOESM7_ESM.pdf]
